# Analysis of cell cycle parameters during the transition from unhindered growth to ribosomal and translational stress conditions

**DOI:** 10.1371/journal.pone.0186494

**Published:** 2017-10-13

**Authors:** Md Shamsuzzaman, Ananth Bommakanti, Aviva Zapinsky, Nusrat Rahman, Clarence Pascual, Lasse Lindahl

**Affiliations:** Department of Biological Sciences, University of Maryland Baltimore County (UMBC), Baltimore, Maryland, United States of America; Texas A&M University College Station, UNITED STATES

## Abstract

Abrogation of ribosome synthesis (ribosomal stress) leads to cell cycle arrest. However, the immediate cell response to cessation of ribosome formation and the transition from normal cell proliferation to cell cycle arrest have not been characterized. Furthermore, there are conflicting conclusions about whether cells are arrested in G2/M or G1, and whether the cause is dismantling ribosomal assembly per se, or the ensuing decreased number of translating ribosomes. To address these questions, we have compared the time kinetics of key cell cycle parameters after inhibiting ribosome formation or function in *Saccharomyces cerevisiae*. Within one-to-two hours of repressing genes for individual ribosomal proteins or Translation Elongation factor 3, configurations of spindles, spindle pole bodies began changing. Actin began depolarizing within 4 hours. Thus the loss of ribosome formation and function is sensed immediately. After several hours no spindles or mitotic actin rings were visible, but membrane ingression was completed in most cells and Ace2 was localized to daughter cell nuclei demonstrating that the G1 stage was reached. Thus cell division was completed without the help of a contractile actin ring. Moreover, cell wall material held mother and daughter cells together resulting in delayed cell separation, suggesting that expression or function of daughter gluconases and chitinases is inhibited. Moreover, cell development changes in very similar ways in response to inhibition of ribosome formation and function, compatible with the notion that decreased translation capacity contributes to arresting the cell cycle after abrogation of ribosome biogenesis. Potential implications for the mechanisms of diseases caused by mutations in ribosomal genes (ribosomopathies) are discussed.

## Introduction

Ribosome biogenesis and cell cycle progression are both controlled by complicated networks. Both processes feature hierarchical waves of proteins that have been studied extensively in *Saccharomyces cerevisiae*. The assembly of yeast ribosomes from the primary rRNA transcript and 79 ribosomal proteins (r-proteins) is orchestrated by more than 200 assembly factors, which facilitate conversion of the primary pre-rRNA transcripts into mature entities, addition of r-proteins to the ribosomal precursor particles, and reorganization of the nascent ribosomes [1–3]. As was originally shown for the assembly of bacterial ribosomes [4–6], the binding of r-proteins to precursor particles is hierarchical such that only a subset of proteins binds directly to the rRNA [7]. The binding of these primary proteins generates binding sites for a secondary wave etc. Moreover, the ribosomal genes are constitutively expressed, although the rate of expression varies according to environmental conditions [8–11]. The time to build a ribosome in yeast is relatively short (about 10 minutes) [12] compared to the doubling time (about 90 minutes in rich glucose medium), but since the cell needs a large number of ribosomes in order to make sufficient protein for a new cell within a doubling time [13], a cell is building thousands of ribosome in parallel.

The cell cycle is also orchestrated by successive hierarchical waves of proteins, which execute the progression through the functional cell cycle stages [14, 15]. However, unlike the ribosomal genes, the cell cycle genes are not expressed constitutively. Rather, proteins with specific functions, such as DNA synthesis or formation of mitotic structures, are expressed in each successive phase of the cell cycle. On the other hand, the cell cycle is similar to the ribosome assembly in that morphopoetic factors orchestrate the assembly of multi-protein complexes [16, 17]. Another difference is that the cell cycle, per definition, lasts through a full doubling time, while ribosome assembly is repeated about 200,000 times in each cell cycle [13].

Interactions between ribosome and cell cycle networks balance cell proliferation and ribosome production, a fundamental of equilibrating the need for protein synthesis capacity with the expense of making ribosomes [13, 18, 19]. Examining the communication between the two regulatory networks is important not only for understanding the regulation of cell proliferation, but also for comprehending the mechanisms for congenital diseases caused by mutations in genes for ribosomal proteins (r-proteins) and assembly factors (ribosomopathies) [20–24]. Current evidence suggests that incomplete ribosomal assembly leads to turnover of nascent ribosomes and accumulation of extraribosomal r-protein complexes that interfere with the turnover of p53 in metazoans [24–28]. However, mechanisms independent of p53 also appear to contribute to these calamities [25, 29–31].

Many mechanisms for both ribosome biogenesis and cell cycle are conserved from yeast to mammals, although details have evolved. However, yeast has no known equivalent of p53 and is therefore well-suited for investigating the interaction between ribosome formation and cell cycle progression in the absence of p53. Indeed, the mechanisms for several ribosomopathies have recently been modeled in yeast by mutating yeast equivalents of human ribosomopathy genes [32–34] supporting the competency of yeast as a disease model for ribosomopathies.

Given that ribosome biogenesis is the most energy consuming process of a cell and involves numerous proteins and RNA molecules, inhibition of ribosome biogenesis (“ribosomal stress”) has multiple systemic effects on the cell. One end result of these effects is cell cycle arrest, which has been observed in organisms from yeast to metazoans [35–41]. While the cells have been investigated after the arrest stage has been reached, the transition from normal cell cycle to arrest after inhibition of ribosome formation has not been explored. To better understand how cell development changes in response to interference with ribosome formation, we repressed individual r-protein genes and quantitatively documented the kinetics of multiple cell cycle parameters during the transition from uninhibited growth to cell cycle arrest.

Some previous investigations suggested that the ribosome biogenesis per se affects the cell cycle, while others suggested that ribosome proteins or biogenesis factors have extraribosomal functions, and yet others suggested that protein synthesis capacity is the crucial parameter [41–44]. Hence, we have also repressed the gene for Elongation Factor 3 (eEF3) to determine whether the cell cycle is affected in response to inhibiting ribosome function (“translational stress”). Our results show that within one-to-two hours of inhibiting r-protein or eEF3 synthesis, budding declined, and after two-to-four actin was depolarized and cell separation after mitosis was delayed. This similarity suggests that declining translation capacity contributes to arresting the cell cycle after abolishing ribosome biogenesis.

## Materials and methods

### Nomenclature

We use the universal nomenclature for r-proteins [45], and indicate the classic yeast name in parenthesis the first time a protein is mentioned.

### Strains and growth conditions

Yeast strains were derived from BY4741. In the Pgal-uS4, Pgal-L4B, Pgal-uL30, Pgal-eL43A, Pgal-Pwp2, and Pgal-Nop7 strains the chromosomal gene(s)s for the r-proteins uS4 (RpS9), uL4 (RpL4), uL30 (RpL7), and eL43 (RpL43), or ribosomal assembly factors Pwp2 and Nop7, respectively, were deleted and a CEN plasmid carrying *RPS4A*, *RPL7A*, *RpL43A*, PWP2, or NOP7, respectively, transcribed from the GAL1/10 promoter was introduced [41, 46, 47]. P*gal*-*TEF3* was constructed by replacing the endogenous promoter with the *GAL1/10* promoter using homologous recombination in BY4741 using KanMX for selection. We note that yeast harbors a second gene for eEF3 called HEF3, which, however, is not expressed [48]. Indeed, the growth curves after repression of the TEF3 gene were the same whether HEF3 was deleted or not (S1 Fig).

Tub1 and Spc42 genes were C-terminally tagged with GFP or mcherry, respectively, in the indicated strains using homologous recombination. GFP-Ras2 and Ace2-GFP were subcloned from pRS315-GFP-Ras2 [49] (a gift from Dr. E. Bi) and pELW754 ACE2-GFP::LEU2 [50] (a gift from Dr. EL Weiss), respectively, into PRS316(URA3) and are expressed from their native promoters. *Sec63*-GFP [51] was introduced on the plasmid ps1622 purchased from Addgene. RFP-PUS1 was subcloned from pRS313-RFP-PUS1 (Han et al. 2007) into pRS402 (ADE2) and integrated into the chromosome at the PUS1 location.

Cultures were grown asynchronously in 1% yeast extract, 2% peptone, 2% galactose (YPGal) at 30°C until mid–log phase (OD_600_ 0.8–1.0, corresponding to 1.5–2 × 10^7^ cells/ml) and then diluted 1:10 into 1% yeast extract, 2% peptone, 2% glucose (YPD). Alternatively, glucose was added to a galactose culture to a final concentration of 2%. Cells were harvested before and at the indicated times after the shift to glucose medium. Cultures were diluted as necessary with pre-warmed media to keep the OD_600_ <1.0 using a Hitachi U1100 spectrophotometer (Hitachi High-Technologies Corporation, Japan).

### Western analysis

Western analysis was performed as described previously [52]. Polyclonal anti-eEF3 (1:10,000 dilution) was purchased from Kerafast Inc, Boston, MA, USA. Polyclonal antiserum from rabbits against r-protein uL18 (L5) was prepared for our lab by Covance Research Products, Denver, CO, USA, using as antigen a synthetic peptide corresponding to the N-terminal 17 amino acids of uL18.

### Confocal microscopy

Cells were fixed for 30 min by adding 3% paraformaldehyde (final concentration) to one OD_600_ unit of cells. Cells were then collected by centrifugation at 5000 rpm for 5 min and washed twice with 0.1 M potassium phosphate pH 7. Cells were then sonicated before being viewed under a Leica TCS SP5 confocal microscope equipped with LAS AF Lite Software using the 63× oil immersion lens. The filter was set at 488 nm for the excitation and 525 nm for the emission for GFP, and at 569 nm for the excitation and 610 nm for the emission for mCHERRY.

### Phalloidin staining

Cells grown at 30°C were fixed as above for 15 minutes. One OD600 unit (1X10^7^ cells) was then spun down at 3000 RPM for 5 minutes using tabletop microcentrifuge. Cells were resuspended and incubated in 0.1 M potassium phosphate pH 7 containing 3% paraformaldehyde for 1hr, then washed twice with 0.1 M potassium phosphate pH 7. Finally, cells were pelleted and resuspended in 25 μl of 3.3 μM phalloidin conjugated with Rhodamine (ThermoFisher) dissolved in PBS with 0.1% Triton X-100. Cells were incubated for 30 min in dark with rocking, then washed twice with PBS and prepared for confocal microscopy.

### Zymolyase digestion

One OD_600_ of culture was collected and fixed with 4% paraformaldehyde for 1hr. Cells were then washed twice and resuspended in 0.1 M potassium phosphate pH7/1M Sorbitol solution containing 0.2 mg/ml zymolyase (US Biological Science). Cells were incubated at 37°C for 1hour, then washed twice with 0.1 M potassium phosphate pH7/1M Sorbitol solution and prepared for brightfield microscopy.

### Sucrose gradient centrifugation

Cells were grown in media (YPGal or YPD) appropriate for the undepleted and depleted conditions (see above). Quick-chilled cells were then spun down resuspended in PA Lysis Buffer (0.05M Tris HCl at pH 7.5, 30mM MgCl2, 0.1M NaCl and 200 μg/mL heparin) and repelleted. The cell pellet was resuspended in 1.25 mL of PA Lysis Buffer and transferred to a tube containing 3g of glass beads (0.5 mm diameter) and vortexed eight times for 30 seconds at 4°C interrupted by cooling in ice water for 1 minute, and diluted with 1.2 mL PA Lysis Buffer. The lysate was spun in 4°C twice at 10,000 g and the supernatant was transferred to a new precooled tube each time. Twenty A^260^ units of lysate were loaded onto a 10–50% sucrose gradient bed. The gradients were spun at 40,000 rpm for 4 hours at 4°C in a SW40 Beckman rotor. Gradients were collected using an ISCO Foxy Jr. sucrose gradient collector pumping at 1 ml/min.

## Results

### Ribosome content during repression of r-proteins and translation factor 3

We used yeast strains in which the only gene for a particular protein is transcribed from the *GAL1/10* promoter. These strains are referred to as “Pgal-Protein Name”. Changing the carbon source from galactose to glucose represses transcription of the gene expressed from the galactose promoter, which results in a gradual decrease in the growth rate (S1 Fig). Northern analysis previously verified that the transcription of r-protein genes under control of the galactose promoter is completely repressed in glucose medium, while transcription of other r-proteins transcribed from the native promoters continues [41]. One hour after the switch to glucose, the abundance of the protein encoded by the gal-controlled gene (r-protein_i_/total protein) was reduced by about 20% and is virtually absent after 8 hours (B. Gregory, A. Lescure and L. Lindahl, manuscript in preparation). Since each ribosome contains one copy of each r-protein, this implies that the ribosome concentration (ribosomes/total protein) also was decreased by 20% one hour after glucose repression of the Pgal-expressed r-protein gene.

**Fig 1:**
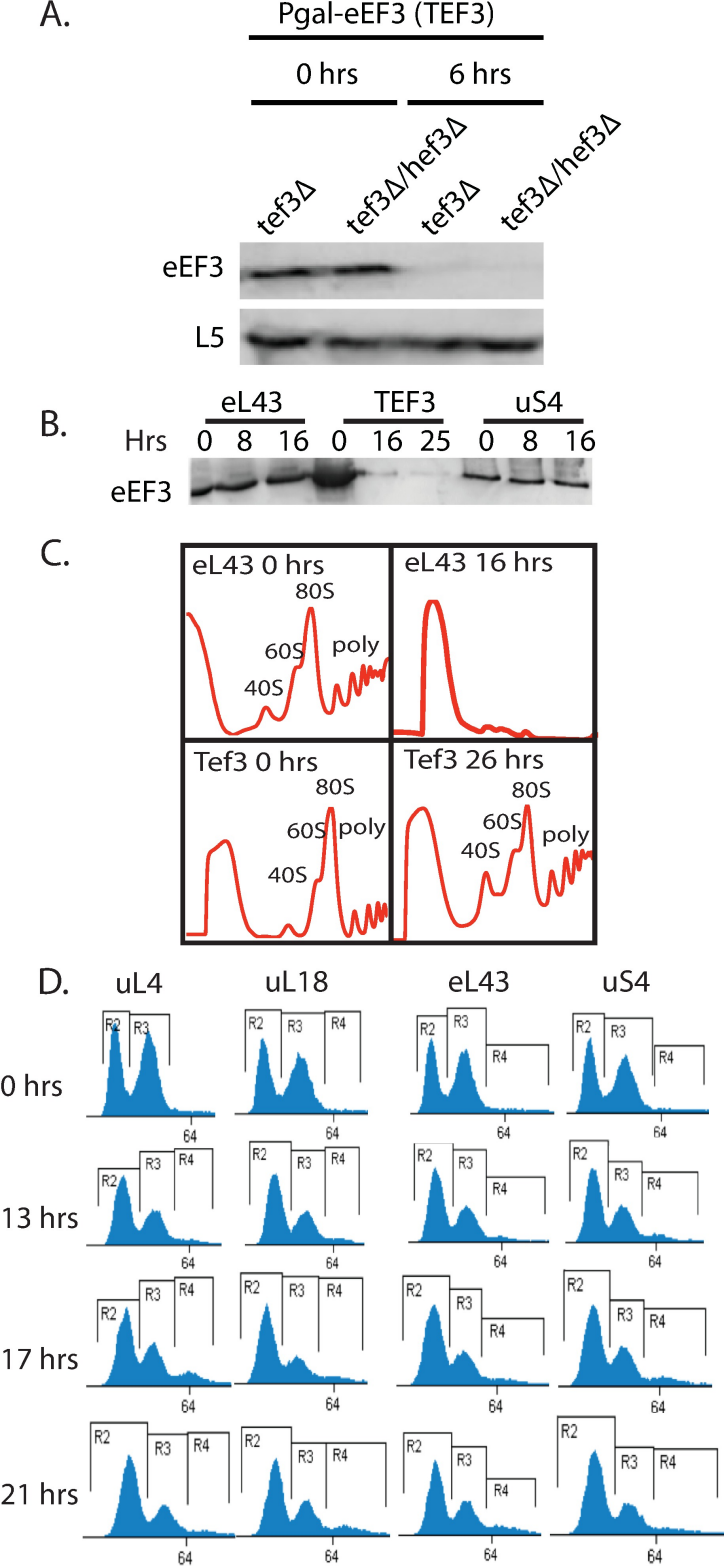
Comparison of repression of r-proteins and translation elongation factor 3 (eEF3). Strains in which uL4, uL18, eL43, uS4, or eEF3 synthesis is under control of the *GAL1/10* promoter were grown in galactose medium and shifted to glucose medium for the indicated length of time. (A) Western analysis of eEF3 before and after switching strains expressing eEF3 from the Gal1/10 promoter to glucose medium. The chromosomal gene of TEF3, or both TEF3 and HEF3, were deleted. R-protein uL18 was used as a loading standard. (B) Western analysis of eEF3 before and after glucose repression of the genes for uL4, eEF3, or uS4. (C) Sucrose gradient analysis of extracts prepared before and after repression of eL43 for 16 hours and eEF3 for 26 hours. (D) Flow cytometry (cell number vs. DNA content) of Pgal-uL4B, -uL18, -eL43, and–uS4 growing in galactose or shifted to glucose for the indicated times. Brackets R2, R3, and R4 correspond to 1N, 2N and 3N amounts of DNA, respectively.

Depletion of eEF3 protein in the Pgal-TEF3 strain was verified by western analysis. As seen in Fig 1A, eEF3 was essentially eliminated 6 hours after the switch to glucose medium in both the tef3Δ and the tef3Δ hef3Δ strains (the strain carrying a single deletion of TEF3 was used in the remaining experiments). In contrast, the eEF3 level remained unchanged after repression of the eL43 or uS4 genes (Fig 1B). To determine if abrogation of eEF3 synthesis affected ribosome content we compared sucrose gradients of equal numbers of A^260^ units of total cell lysates of Pgal-eEF3 and Pgal-eL43 before and after switching from galactose to glucose medium. Twenty-six hours after repression of *TEF3* the ribosome level was not significantly affected. In contrast, the ribosome content in Pgal-eL43 was nearly obliterated after 16 hours in glucose medium (Fig 1C). Thus, as expected, depletion of eEF3 did not decrease the ribosome content, while the cell ribosome content decreased after repression of r-protein synthesis. Moreover, the fraction of ribosomes in polysomes increased after abolishing the eEF3 synthesis, presumably because ribosome movement on the mRNAs was decreased or blocked. Similar enhancement of the polysome fraction is seen after inhibition of translation elongation with cycloheximide [53].

### The number of daughter cells attached to mother cells is dynamic

We previously showed that mother cells with two or three attached buds or daughter cells accumulate after the shift from galactose to glucose medium of some Pgal-r-protein strains. This effect is not seen after the same shift of carbon source with the parent strain (BY4741) and can thus be ascribed to the repression of a subset of r-protein genes [41]. These cell complexes generate a peak with more than 2N DNA equivalents in flow cytometry (“3N” peak). Since the growth kinetics after shifting different Pgal-r-protein strains to glucose medium differed somewhat, we suspected the 3N-phenotype might depend on the time elapsed after the shift. Accordingly, we performed flow cytometry on several different Pgal-r-protein strains at different times after a shift from galactose to glucose. As previously reported [41], a 3N peak was observed 17 hours after the cessation of uL4 (L4) synthesis, but we now found that the peak faded by 21 hours (Fig 1D). Other strains, in which we previously did not see a 3N peak at 16 hours (Pgal-uL18 (L5), eL43 (L43), and uS4 (S9)), formed small, but significant, 3N peaks that were also temporary and presented at different times (Fig 1C). Thus, at least for these strains, there is a dynamic generation and resolution of di- and tri-budded mother cells and it is the timing and not the strain that determines if a 3N peak is observed in the flow cytometer.

### The number of mitotic spindles decline and long astral microtubules accumulate

To document the developmental changes of yeast cells after abrogating the synthesis of r-proteins or eEF3, we aimed to quantify developmental stages based on specific molecular markers. We first looked qualitatively at spindles and spindle pole bodies (SPBs), the yeast functional equivalent of the centrosome, which are useful “clocks” for the cell cycle. During normal development, cells appear from the G1 to S phase transition with one bud and one SPB. As the bud grows, the SPB is duplicated. The two SPBs first move to the budneck and subsequently become the endpoints of the emerging spindle. During mitosis the SPBs move to opposite ends of the mother-daughter cell axis and until they are at opposite ends of this axis and connected with a long spindle in anaphase. After the cell division is completed each cell has an SPB associated with a long astral microtubule.

**Fig 2:**
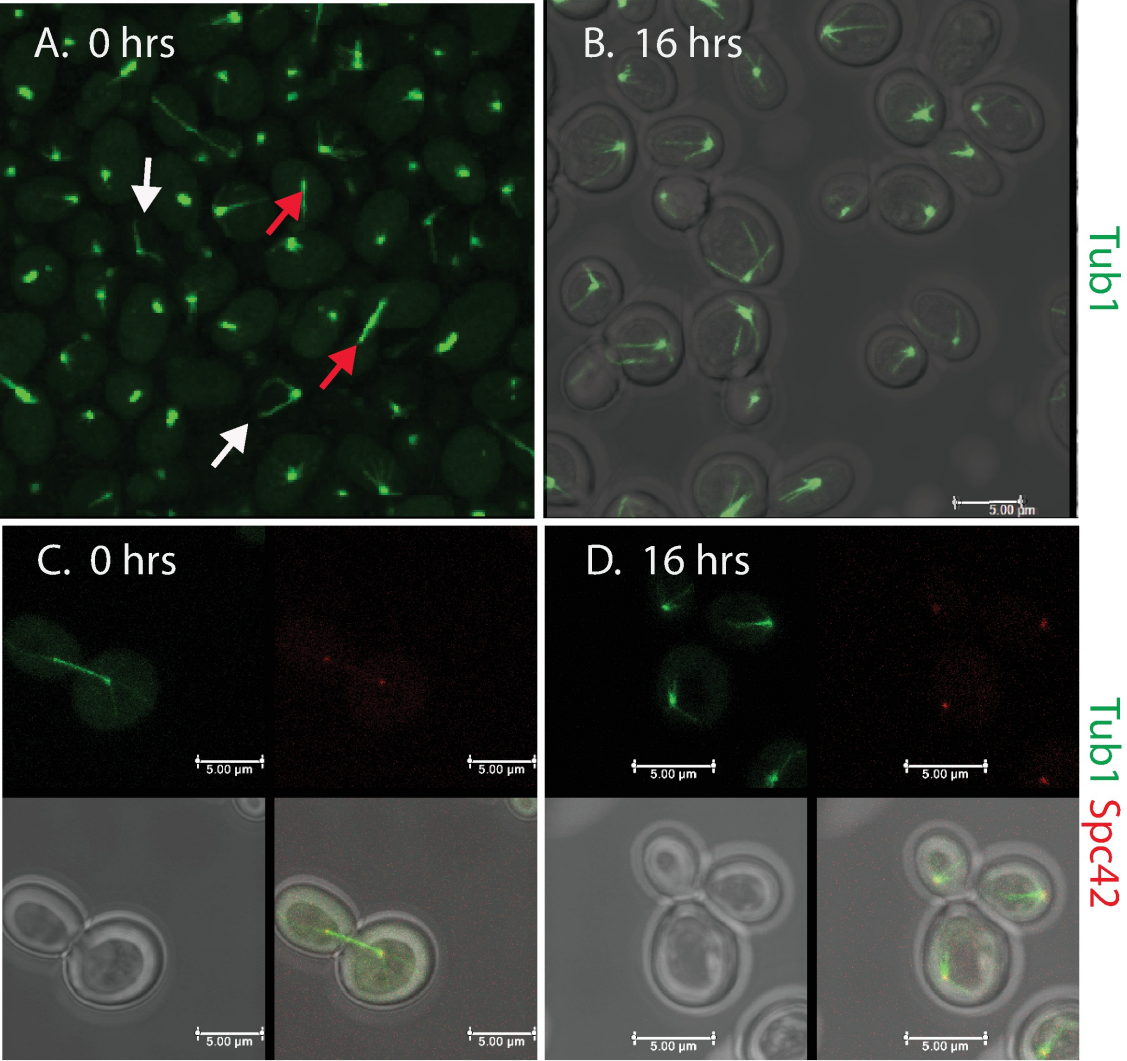
Dynamics of spindle and spindle pole body (SPB) during ribosomal stress. The Pgal-eL43 strain tagged with (A-B) Tub1-GFP or (C-D) Tub1-GFP and Spc42-RFP were grown in galactose (A and C) and shifted to glucose for 16 hours (B and D). (A) Spindle structures in cells growing in galactose. The white arrows in point to the long astral microtubules and red arrows point to anaphase spindles. (B) Merge of bright field and Tub1-GFP after 16 hours in glucose medium. Note the long astral microtubules in most cells. (C-D) Merges of Tub1-GFP and Spc42-RFP in cells growing in (C) galactose or (D) shifted to glucose for 16 hours. The top left shows Tub1-GFP, top right shows Spc42-RFP, bottom left shows the brightfield image, and right bottom shows the merged images.

We first introduced a GFP tagged tubulin gene and inspected large numbers of cells by confocal microscopy. Fig 2A illustrates examples of confocal images of cells from the Pgal-eL43 strain. During growth in galactose medium we observed all stages of tubulin assemblies that collectively describe the normal cell cycle (Fig 2A). Since the cells were not synchronized, the frequency of specific cell cycles stages was low. For example, long anaphase spindles were seen in 8–14% of the cells, and few cells contained a long astral microtubule, as expected, because astral microtubules only exist in a fraction of the cell cycle, and furthermore, the astral microtubules expand and contract in G1 during normal growth [54, 55]. Sixteen hours after repression of r-protein synthesis virtually all cells contained long astral microtubules, but no long spindles were seen (Fig 2B). The same pattern was seen after inhibiting the synthesis of other ribosomal proteins as exemplified by an image of Pgal-uL4 (S2 Fig). We conclude that the great preponderance of these cells were in G1 phase. Furthermore, mother cells with two, or even three, attached buds or daughter cells accumulated (see Fig 2B for Pgal-eL43, and S2 and S3 Figs for Pgal-uL4 and–uS4, respectively).

For further characterization we added an RFP tag to the chromosomal *SPC42* gene, which encodes a protein in spindle pole bodies (SPB). During growth in galactose, SPB dynamics was normal: single SPBs were seen in mothers with small buds, while mothers with larger buds harbored duplicated SPBs in the budneck or migrating towards the poles in each cell (Fig 2C). Moreover, SPBs were attached to astral microtubules at each end of long spindles in late stages of mitosis (Fig 2C). After repressing of eL43 synthesis for 16 hours virtually all cells harbored an SPB attached to a long astral microtubule compatible with G1 arrest and suggesting that the balance between growth and contraction of the astral microtubule was shifted in favor of longs astral microtubules (Fig 2B and 2D, and S2 Fig). Quantification of spindle dynamics and SPB positions is presented below in combination with data from analysis of membrane ingression.

### Membrane ingression continues during inhibition of ribosome formation and function

The absence of mitotic spindles in connected mother and daughter cells suggested that mitosis is completed, but since many daughter cells did not separate from their mother cells, we asked whether the attached mother and daughter cells had undergone cytokinesis with membrane ingression. During normal growth cells separate after telophase within a short time [56].

**Fig 3:**
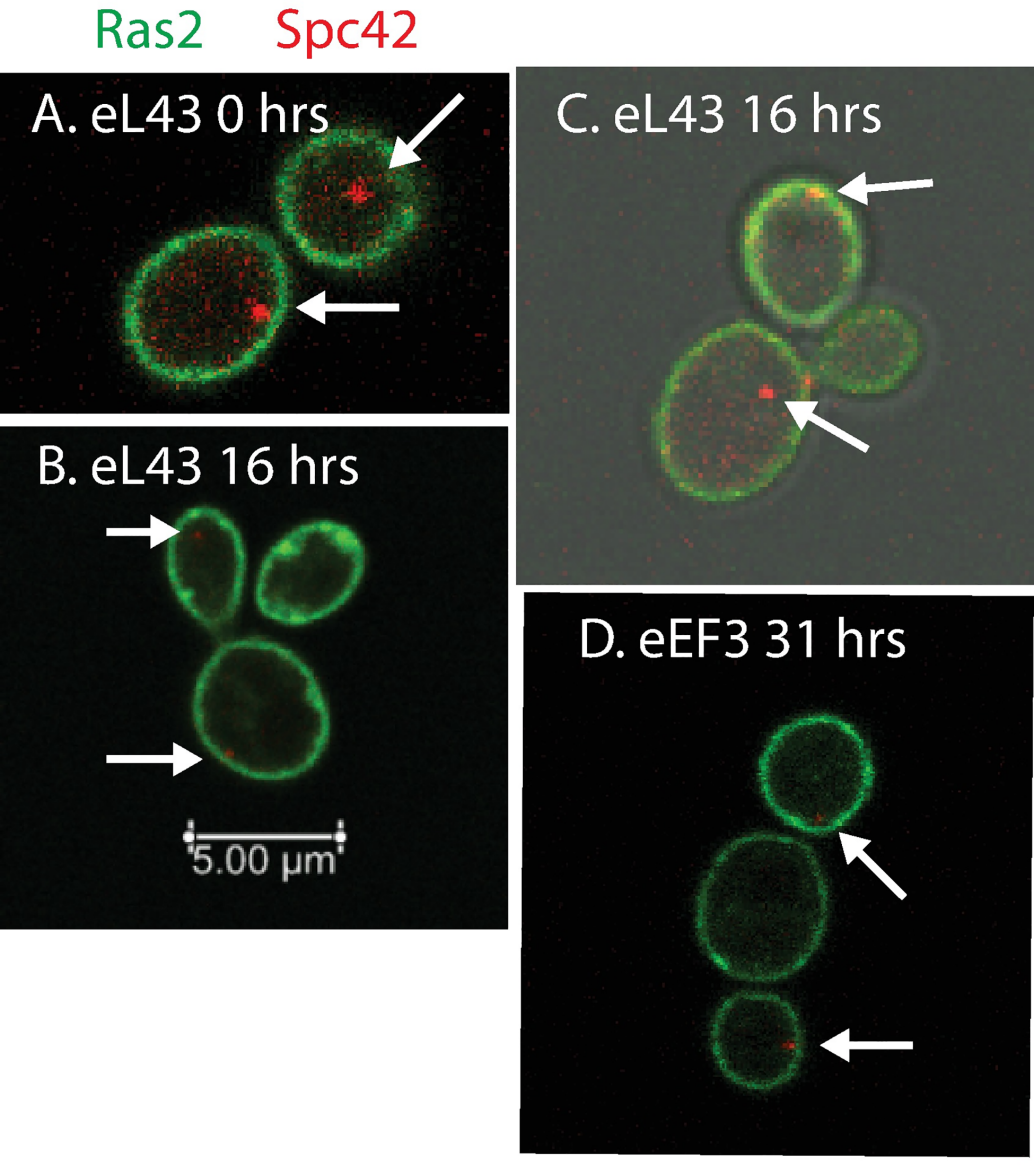
Dynamics of cell membranes during ribosomal and translational stress. The plasma membrane protein Ras2 was tagged by GFP and the SPB protein Spc42 was tagged by RFP. (A) Pgal-eL43 growing unhindered in galactose medium. (B-C) Mother-daughter complexes of Pgal-eL43 after 16 hours in glucose medium. (D) Mother-daughter complexes of Pgal-eEF3 synthesis after 31 hours in glucose medium. Arrows point to SPBs.

To follow membrane ingression, we marked the cell membrane in Pgal-eL43 and Pgal-TEF3 by introducing a GFP tagged gene for Ras2, a component of the plasma membrane. Furthermore, SPBs were marked with Spc42-RFP. In galactose cultures we only saw complete membrane ingression in separated cells or in mother cells connected with a single daughter cell (Fig 3(A)), showing that daughter cells separate from mothers with little delay after completion of membrane ingression, as expected for normally growing cells [56]. After repressing either the synthesis of eL43 (Fig 3B and 3C), eEF3 (Fig 3(D)), or uS4 (S3 Fig), we saw an increasing number of mother cells with two attached daughters, one or both of which had completed membrane ingression. This development is quantified as a function of time below. Importantly, membrane ingression was always complete for one daughter, before the next bud formed. Thus separation of many daughter cells from their mother cells, was delayed by a doubling time, or more in the case of three attached daughters/buds, after cytokinesis was completed. We refer to mother cells associated with at least one daughter with a fully closed membrane as “mother-daughter complexes”. Cells, which are not attached to another fully developed cell, will be referred to as “single cells”, even though these cells may have a bud. Strikingly, daughter cells in the mother-daughter complexes never formed buds of their own (bud-on-bud) after repression of eL43 or uS4 synthesis (202 daughter cells examined), indicating that daughter cells were arrested while many mother cells still had the capacity form new buds. In the Pgal-eEF3 strain bud-on-bud was seen in rare instances (8 of 138 buds examined), suggesting that the arrest of daughter cells was somewhat slower after repression of eEF3 synthesis.

**Fig 4:**
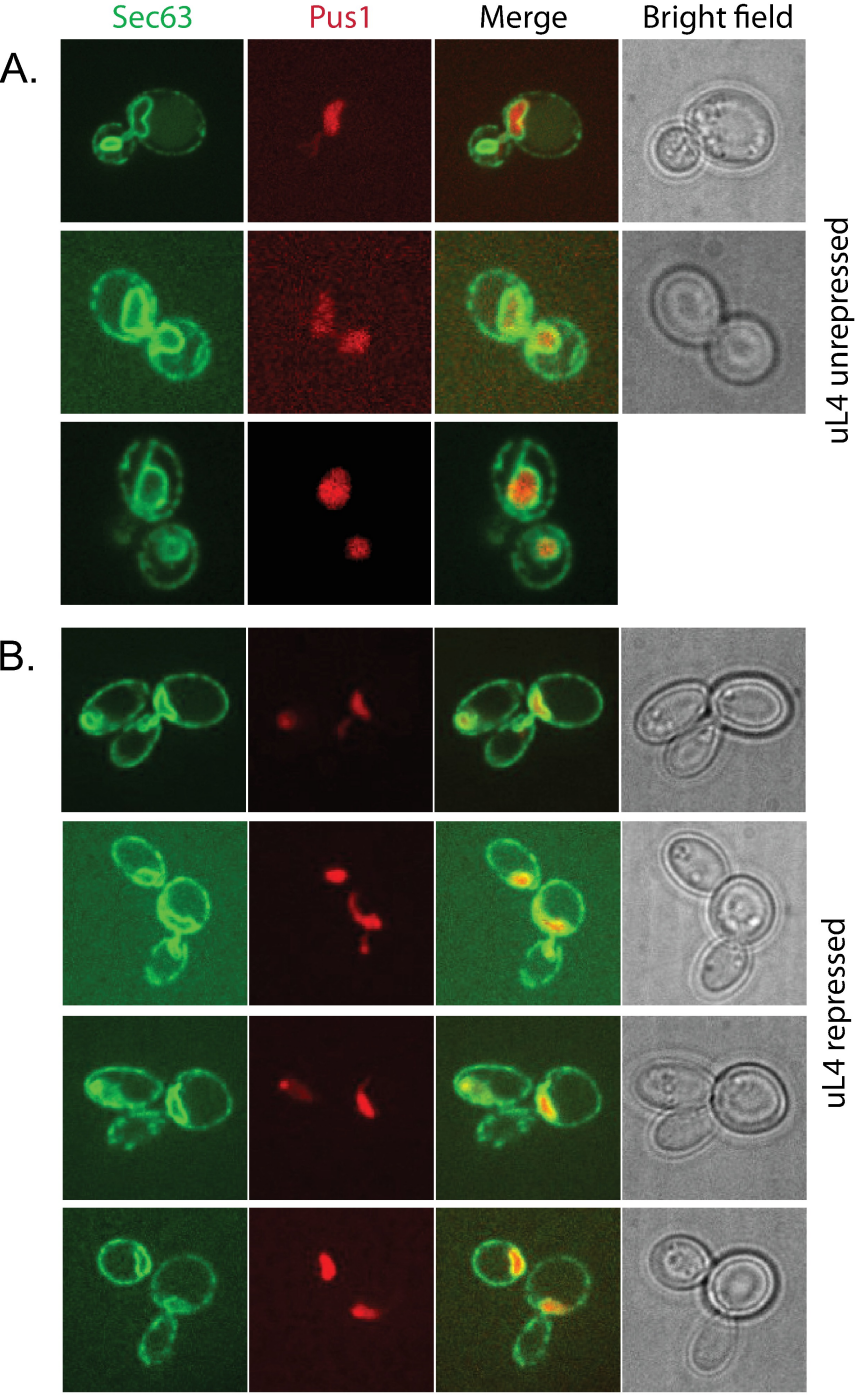
Stages of migrating nucleus in the budneck before and after repression of uL4 synthesis. The Pgal-uL4B strain was modified with Pus2-RFP as a marker for nucleoplasm and Sec63-GFP as a marker for nuclear and cell membranes. (A) Stages of mitosis during growth in galactose. (B) Cells after growth in glucose for 15 hours. Mothers are associated with one post mitotic daughter surrounded by a complete plasma membrane and containing its own nucleus. A second bud has formed at each of the mother cells and the nucleus migrating to the second daughter is seen in the budneck.

To expand the number of strains analyzed for membrane dynamics we also investigated the cell development after abolishing the synthesis of uL4 (L4). To visualize movement of the nucleus movements, we introduced a gene for GFP-tagged Sec63, a protein in both the nuclear and plasma membrane. Moreover, we RFP-tagged Pus1, a pseudouridine synthase localized to the nucleoplasm [57]. Normal mitosis was observed prior to cessation of uL4 synthesis (Fig 4A), but 16 hours after repressing uL4 synthesis mother-daughter complexes had accumulated (Fig 4B). In some cases separation of nuclei and membrane ingression were both complete. In other cases, the plasma and nuclear membranes were complete for one daughter cell, while the plasma membrane was open between the mother cell and the second bud, and the daughter nucleus was stretched through the budneck (Fig 4B). We did not see open membranes between mother cells and both buds and we did not see new buds on daughter cells, confirming the pattern during repression of eL43 synthesis.

In summary, cytokinesis is completed during both ribosomal and translational stress, but cell separation is delayed resulting in mother cells with two daughter cells, or a daughter and a bud. Furthermore, the development of the first, second, and sometimes third bud at a mother cell was sequential, and nuclear migration in each of these mitoses appeared identical to the mitosis prior to onset of ribosomal stress. These results show that the 2N and 3N peaks in flow cytometry analysis may contain post mitotic cells that have not yet separated. Consequently, the distinction between M and G1 phase cannot be determined accurately based on flow cytometry only, but requires microscopic analysis of membrane ingression.

### Cell development changes after short periods of inhibiting ribosome formation or function

**Fig 5:**
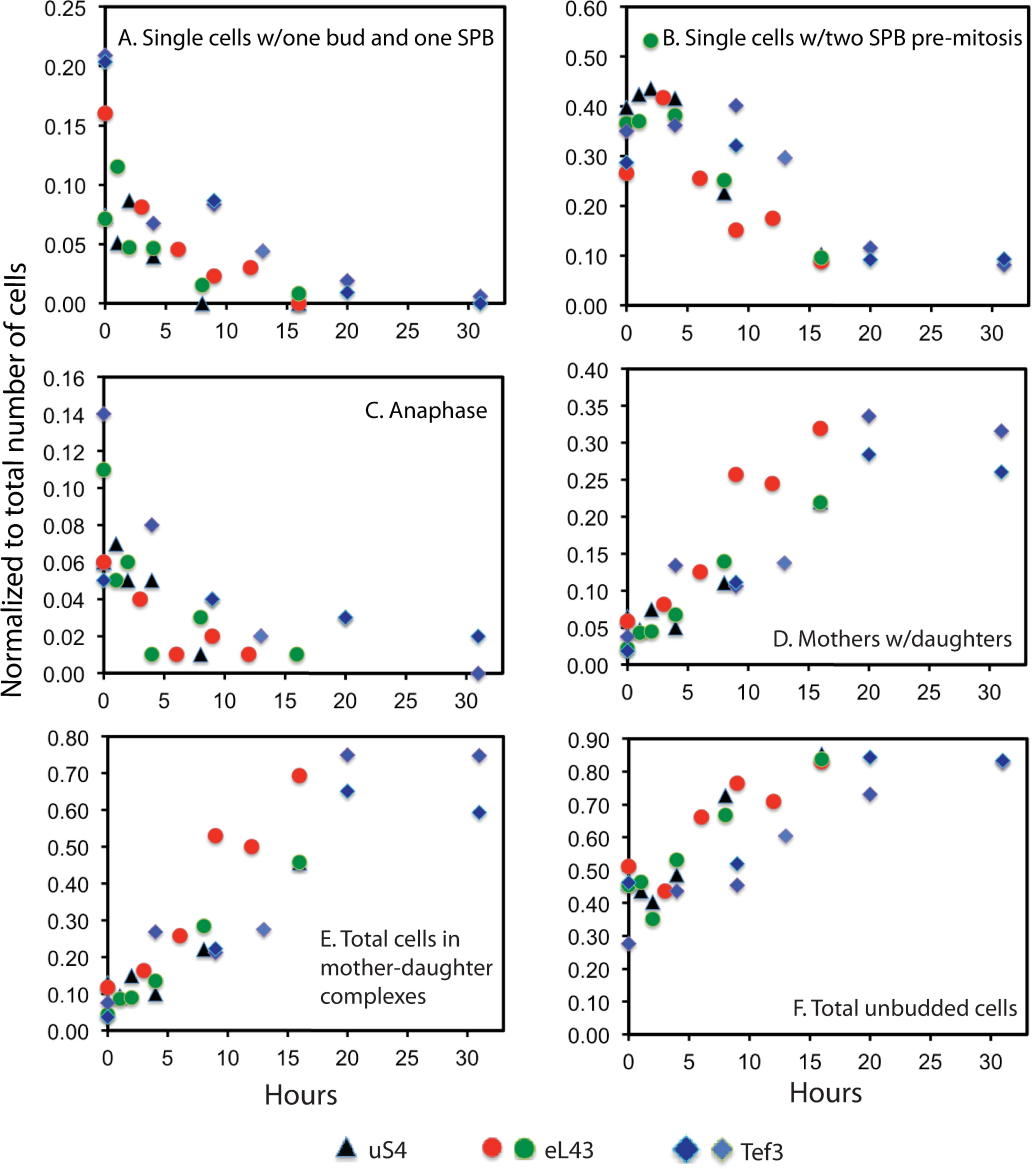
Quantification of cell cycle stages classified by spindle and membrane structure, and position of SPB(s). Data were obtained from classifying and counting cells on field images collected at different times beginning one hour after the repression of the genes for uS4, eL43, or eEF3. A cell is counted as an individual cell, if it is surrounded by a completed plasma membrane, whether it is in mother-daughter complexes or a single cell. Cells in each classification are normalized to the total number of cells. (A) Single cells (i.e. cells not in mother daughter complexes) with one bud and one SPB. (B) Single cells with two SPBs with no spindle or spindles shorter than an anaphase spindle. (C) Anaphase cells with two SPBs, one at each end of the mother-daughter axis and connected by a long spindle. (D) Mother cells with attached daughters surrounded by a complete plasma membrane, i.e. “mother-daughter complexes”. (E) Total number of cells surrounded by a plasma membrane in mother-daughter complexes. F. Total number of unbudded cells (single cells or in mother daughter complexes). The number of cells counted for each time point was 101–295. (See S1 Table for raw cell counts.) Blue triangles: repressed uS4 synthesis; red and green circles: biological replicates of repressed eL43 synthesis; light and dark blue diamonds: biological replicates of repressed eEF3 synthesis.

The images in Figs 2–4 show the status after 15–16 hours of ribosomal or 31 hours of translational stress. To document the kinetics of the evolving changes in cell configurations during the transition from unhindered growth to stress-induced arrest, we collected field images of cultures at different times after imposing stress, beginning one hour after the shift to glucose medium. Cells with different configurations of spindles, SPBs, and membrane ingression were classified, counted, and normalized to total number of cells. Due to the delayed post-mitotic cell separation described above we counted each cell with a complete plasma membrane as an individual cell whether it was in a mother-daughter complex or a single cell. Results are summarized in Fig 5.

We first note that the change in the abundance of cell cycle stages is the same after inhibition of the synthesis of the 40S protein uS4 or the 60S protein eL43, but the kinetics is delayed after inhibition of eEF3 synthesis relative to abrogating r-protein synthesis (Fig 5). The abundance of single cells with one bud and one SPB (i.e. in early stages after START) begin declining with one-to-two hours after imposition of stress and are essentially absent by 16 hrs (Fig 5A), suggesting that budding process is inhibited a short time after imposition of stress. The fraction of cells with two SPBs, but no long spindle (i.e. pre-anaphase) also declined, albeit later than cells with only one SPB (Fig 5B). Unexpectedly, cells in anaphase (two SPBs at opposite ends of the mother-daughter axis connected with a long spindle) decreased with kinetics similar to the kinetics of single cells with one bud and one SPB, and was not delayed as was the decline of cells with two SPBs (Fig 5C), suggesting that the relative time cells spent in each cell cycle phase was changed after the shift from galactose to glucose medium.

Accumulation of mother-daughter complexes, indicating a delay of cell separation, began two-to-three hours after the shift to glucose (Fig 5D). These complexes eventually accounted for about 60% of the cells after 16 hours of ribosomal or translational stress (Fig 5E). The total number of unbudded cells, including both single cells and mother-daughter complexes, with a complete plasma membrane (i.e. in G1) increased from 40–50% of the cells during uninhibited growth in galactose to 80–90% of the cells after 16 hours of stress (Fig 5F). As with other cell classifications, the accumulation of G1 cells was somewhat slower in response to repression of eEF3 synthesis than cessation of r-protein synthesis.

The number of cells in mother-daughter complexes never reached 100%, suggesting daughters eventually separate from the mother cells. To test this conclusion we determined size distribution of cells from the forward light scattering in flow cytometry. In galactose medium we found a continuous distribution of sizes as expected for a non-synchronous culture. After the repression of r-protein synthesis, the distribution split into two peaks after repression of r-protein genes (S4 Fig). We interpret these two peaks as single cells and mother-daughter complexes. As previously shown the cell sizes increased after the shift [41].

### Daughter cells are attached to mother by a cell wall tether

**Fig 6:**
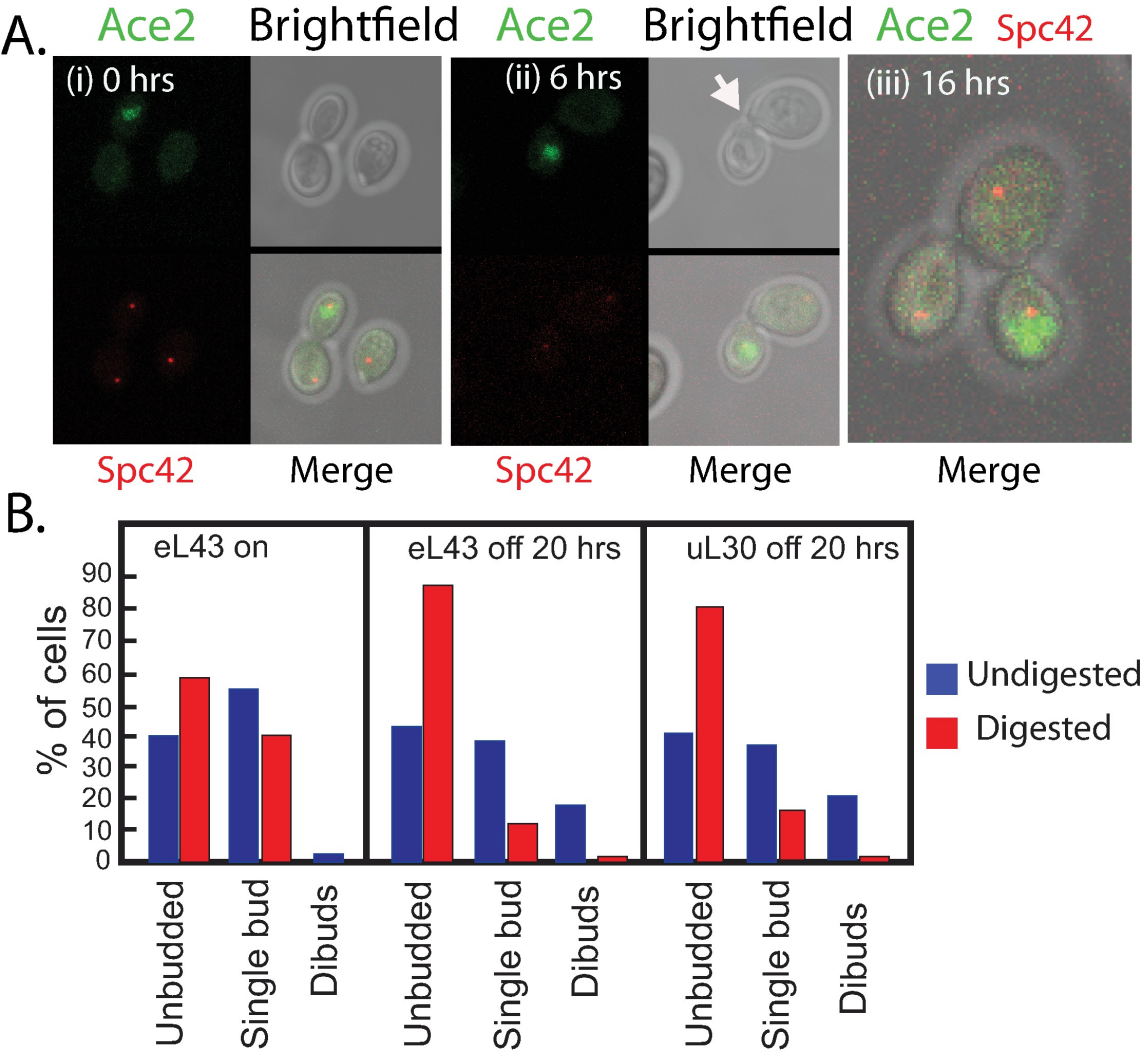
Structure of mother-daughter complexes. (A) Ace2 in Pgal-eL43 was tagged with GFP and Spc42 with RFP. (i) Mother cell with daughter cell during growth in galactose medium. (ii) Mother cells with daughter cell 6 hours after switch to glucose medium. Note the cell wall tether in the bright field image (arrow). (iii) Mother cell with two daughter cells after 16 hours of growth in glucose medium. Note that Ace2 is only in one of the buds. (B) Effect of zymolyase digestion on distribution of single cells, mother cells with bud or daughter, and mother with two daughters or buds in Pgal-eL43 and Pgal-uL30 cultures shifted from galactose to glucose medium for 20 hours. At least 100 cells were counted for each histogram. Raw values for the counts unbudded, single budded and dibudded cells is given in S2 Table.

What binds the mother and daughter cells together? Many images showed a “tether” between mother and daughter, which must be flexible as the angle between mother and daughter cells varied (Fig 6A). We hypothesized that the tether was made of cell wall material. Accordingly, we exposed Pgal-eL43 and Pgal-uL30, harvested before and 20 hours after shift to glucose, to zymolyase, a cell wall degrading enzyme. Cells with one or two buds/daughter cells were counted before and after the digestion. As seen in Fig 6B, approximately 20% of the cells from glucose cultures were dibuds prior to zymolyase treatment, while very few dibuds were seen in the galactose culture (here we do not distinguish between actual buds and post mitotic daughter cells). Essentially, all dibudded mothers in the glucose cultures were separated from at least one bud by the zymolyase treatment (Fig 6B). Moreover, the number of single-budded mothers in the glucose cultures changed from 35–40% before digestion to approximately 15% after digestion and the number of unbudded cells increased from 40% to 80–85%. In comparison, digestion of cells from the galactose culture reduced the number of single budded cells from 55–60% to 40%, a reduction of about 30%, as compared to a 70% reduction in the glucose cultures. Thus the results of the zymolyase digestion are compatible with the notion that mother and daughter cells are indeed held together by cell wall material.

### Ace2 transcription factor is observed in daughter nuclei during ribosomal stress

After completion of cytokinesis cell wall-digestive enzymes like endochitinase and glucanase are expressed in daughter cells and promote cell separation [56]. The transcription of some of these genes is regulated in daughter cells by localization of Ace2 transcription factor to the nucleus [58, 59]. To determine if Ace2 was concentrated in daughter nuclei during ribosomal stress we constructed a Pgal-eL43 derivative harboring GFP tagged *ACE2* and RFP tagged *SPC42*. As shown in Fig 6A, we found Ace2-GFP concentrated in daughter nuclei while SPBs were located at opposite ends of the mother and daughter axis, both before and 6 hours after abrogating eL43 synthesis. After 16 hours Ace2-GFP was concentrated in the nucleus of one daughter cell, but not in the other daughter cell, of mother-daughter complexes, compatible with sequential bud formation described above and the notion that one daughter is in early G1, while the mother has progressed further into G1.

### Actin depolarization begins after short periods of inhibition of both ribosome formation and function

At the beginning of normal budding, actin patches migrate to the impending bud site [14]. After buds have developed the patches become dispersed, and during post-anaphase finally form a ring in the budneck that contributes separation of mother and daughter cells [14, 60, 61].

**Fig 7:**
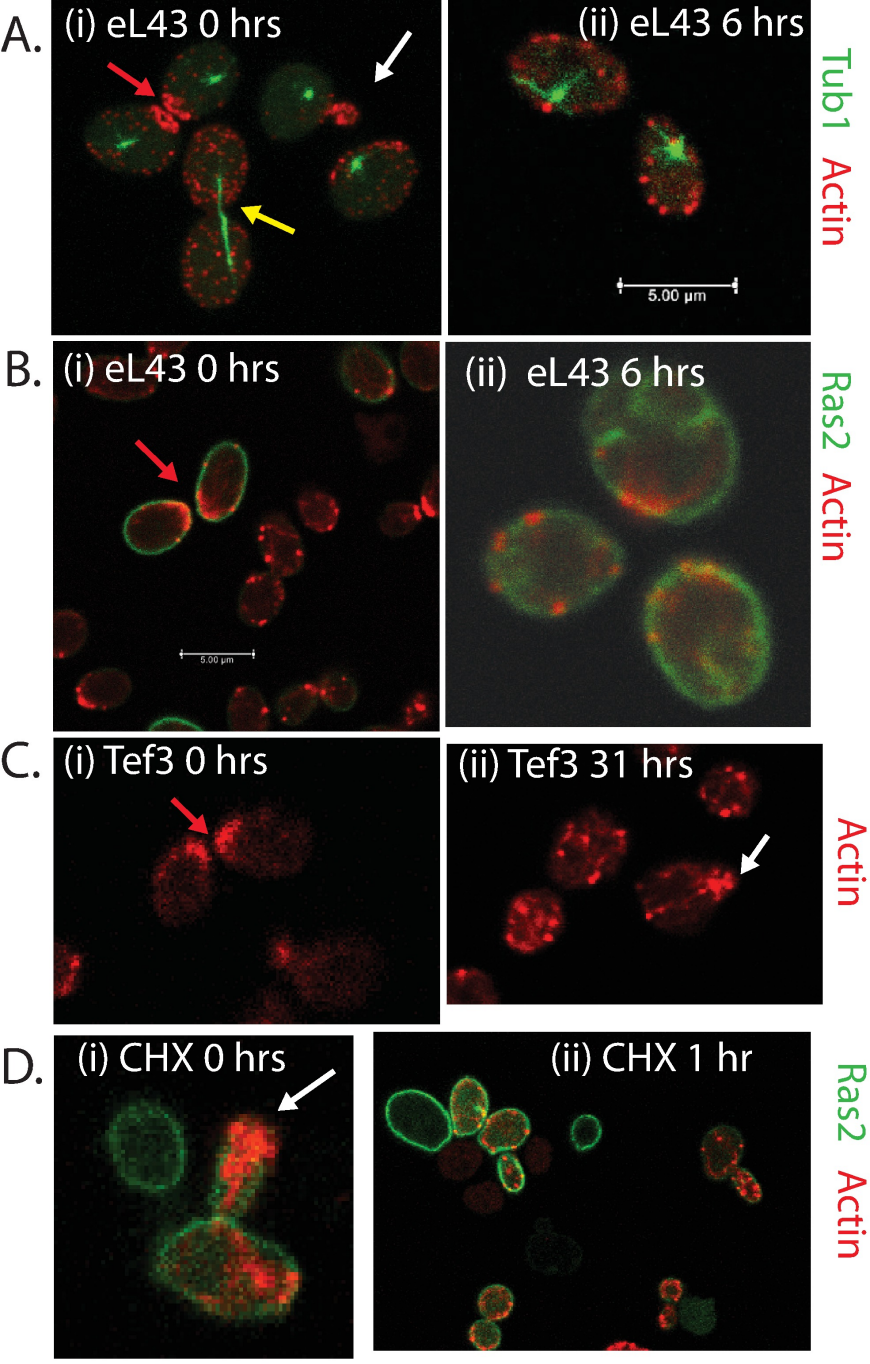
Dynamics of actin patches during ribosomal and translational stress. Actin was stained with rhodamine-phalloidin. (A) Pgal-eL43 tagged with Tub1-GFP was grown in galactose medium and shifted to glucose medium. (i) Cells grown in galactose. (ii) Cells switched to glucose medium for 6 hours. Note the large astral microtubules and the lack of actin rings. (B) Pgal-eL43 strain tagged with GFP-Ras2. (i) Cells growing in galactose. Note the actin rings in cells after membrane ingression. (ii) Cell switched to glucose for 6 hours. Note the lack of actin rings in mother and the two post-mitotic daughter cells. (C) Pgal-eEF3 strain stained with phalloidin. (i) Cells grown in galactose. (ii) Cells after growth in glucose for 31 hours. The image shows a rare example of polarization to budsite. (D) Distribution of actin patches after cycloheximide inhibition of translation. (i) Pgal-uS4 cells grown in galactose without CHX (left), (ii) grown in galactose with 100 ug/ml CHX for 1hr. White arrows point to actin polarized to bud sites. Red arrows show actin rings in mother and daughter cells. The yellow arrow shows a spindle that does not stretch to the ends of the mother daughter axis (pre-anaphase); note that no actin rings are visible at this stage.

It was previously reported that actin is depolarized after repression of the synthesis of the ribosome assembly factors Nop15 or Rrp14 for 14–18 hours [39, 62]. At this time G1 arrest is fully established preventing evaluation of the kinetic relationship between actin polarity and other cell cycle parameters. Accordingly, we correlated the actin patterns with the constellation of other cell cycle parameters. Actin patches were first stained with rhodamine-conjugated phalloidin in Pgal-eL43 harboring a gene for Tub1-GFP or GFP-Ras2. In galactose medium actin patches followed the normal pattern with actin polarized to bud sites in cell with small buds and to the budneck in cells with an anaphase or receding spindles, but not in cells with shorter spindles (Fig 7A(i)). Staining of galactose grown Pgal-L43 tagged with GFP-Ras2 showed that the actin ring at the budneck persisted after membrane ingression was completed (Fig 7B(i)). After cessation of r-protein or eEF3 synthesis actin polarization waned. Six hours after repression of the eL43 gene actin patched were not seen at budsites and rarely at budnecks (Fig 7A(ii) and 7B(ii)). Similar results were seen after repression of the synthesis of the ribosomal assembly factors Nop7 and Pwp2 (S3 and S4 Figs). Actin polarization also decreased after repression of eEF3 synthesis, but more slowly and some polarization to buds was still observed at 16 hours or even 31 hours (compare Fig 7C(i) with 7C(ii)).

**Fig 8:**
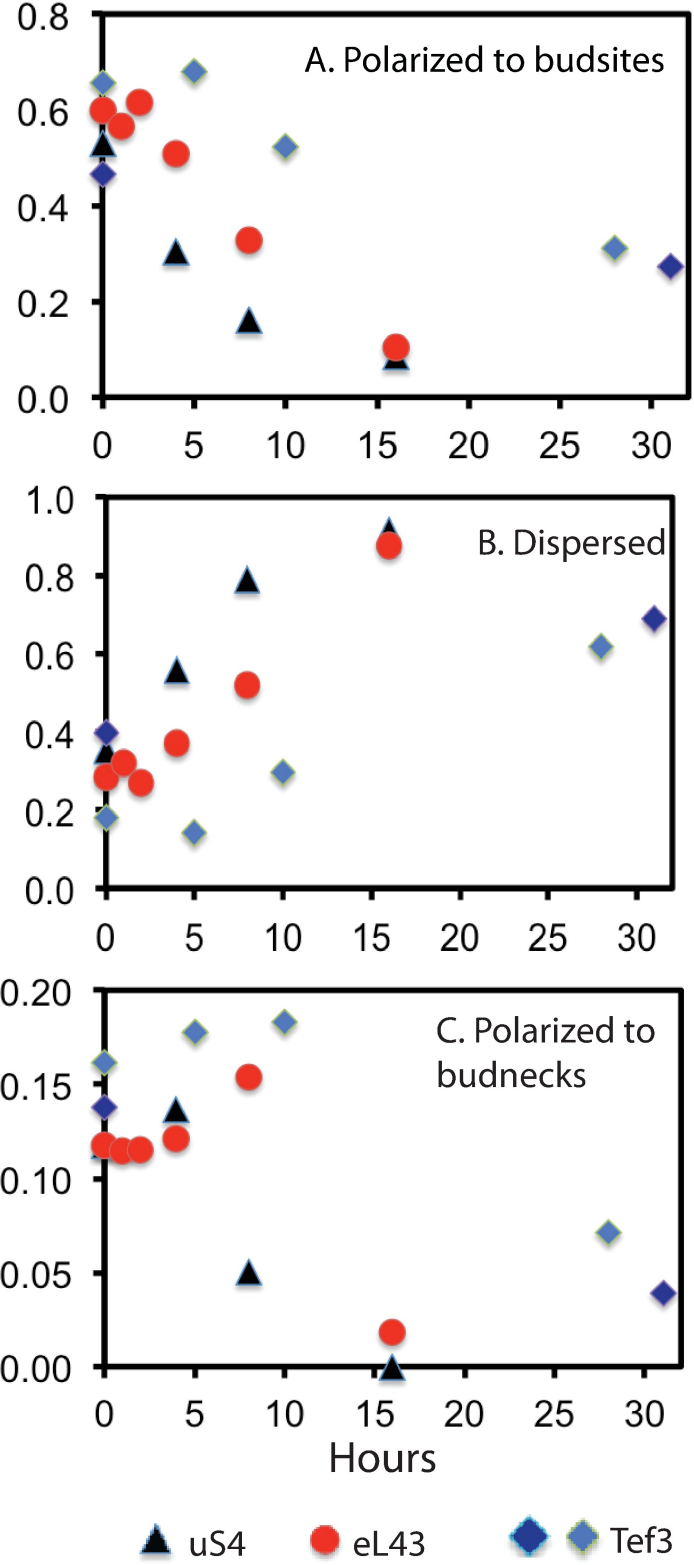
Quantification of polarized and dispersed actin patches. (A-C) The number of cells in each state was normalized to the total number of cells. (A) Polarized to budsite. (B) Dispersed. (C) Polarized to budneck. Between 58 and 446 cells were counted for each strain and time point. Raw data and calculation of the aggregate categories plotted in the figure are shown in S3 Table and an overview of the characteristics of the classifications used is shown in S4 Table.

We quantified actin polarization in Pgal-eL43, Pgal-uS4, and Pgal-eEF3 after imposition of stress. After two-to four hours after repression of eL43 or uS4, actin polarization to bud sites decreased (Fig 8A), while polarization to dispersed actin patches increased (Fig 8B). Polarization to budnecks decreased beginning at about 5 hours after the shift (Fig 8C), suggesting that at least some cells with actin polarized to the bud sites the time of the shift to glucose medium completed the cell cycle. Depolarization after repression of eEF3 synthesis began later and was more gradual compared to r-protein gene repression (Fig 8A–8C). Furthermore, the total number of actin patches per cell decreased about two-fold by 16 hours after repression of r-protein genes, but was unchanged after abrogation of eEF3 synthesis for 31 hours (S7 Fig).

Repression of r-protein genes and the eEF3 gene both result in decreased protein synthesis capacity, suggesting that protein synthesis is important for maintaining actin polarization. To test this hypothesis we stained actin in cells before and one hour after adding 100 μg/ml cycloheximide to a galactose culture. We found that this treatment indeed depolarized actin completely within an hour (Fig 7D), compatible with the notion that active translation is necessary for maintaining actin polarity.

## Discussion

### Cell development changes after short periods of ribosomal or translational stress

While it is well established that inhibition of ribosome biogenesis leads to cell cycle arrest, the transition from uninhibited growth to arrest has not been elucidated, because previous investigations were undertaken 14–18 hours after repressing genes for ribosomal assembly factors, i.e. after the cell cycle arrest is fully established [39, 43, 62]. Here we have shown detailed kinetics of changes to the cell configuration during the transition. The number of budded single cells changed within one-to-two hours of repressing the transcription of a single essential r-protein (Figs 2 and 5) and actin depolarization to bud sites began within two-to four hours. Thus cell cycle progression is modified with little delay after the interference with the expression of an r-protein gene.

### Effects of ribosomal and translational stress

Assembly of mature ribosomes requires a full complement of ribosomal proteins. Blocking the synthesis of an essential r-protein therefore distorts the normal assembly pathways and changes the concentration of ribosomal precursor particles, which might affect signaling pathway(s) to cell cycle progression. However, the dismantling of ribosome assembly also obstructs production of new mature ribosomal subunits. In fact, recent experiments in our lab have shown that one hour after blocking the transcription, the abundance of the r-protein encoded by the repressed gene is decreased by 20% relative to total cell protein, with further reduction for at least seven hours (B. Gregory, A. Lescure and L. Lindahl, manuscript in preparation). Since we have only used genes for essential r-protein genes and ribosomes contain one copy of each ribosomal protein this means that the number of active ribosomes relative to total cell protein is also reduced by about 20% within one hour. That is, abolishing transcription of an r-protein gene reduces the translation capacity within an hour. Consequently, mRNAs must compete for a dwindling number of ribosomes, which may change the ratio between the amounts of different cell cycle proteins synthesized due to differential translation of specific mRNAs.

To determine the specific effect of decreasing the translation capacity on cell cycle progression, we directly changed the translation rate of mature ribosomes by repressing the gene for Translation Factor eEF3 (TEF3). This reduced the concentration of eEF3 to background levels within six hours, but has no detectable effect on the ribosome concentration (Fig 1). The changes in cell configurations after abrogation eEF3 synthesis are essentially the same as those seen after stopping r-protein synthesis (Fig 5), demonstrating that protein synthesis capacity by itself affects the cell cycle. This agrees with the fact that the cell cycle is arrested not only after repression of genes for r-protein and ribosome assembly factors, but also by mutations in numerous genes for translation initiation factors, tRNA synthetases, tRNA, and rRNA modification enzymes [38]. The large number of mutations in translation initiation factors with effects on the cell cycle could suggest that the arrest is caused specifically by a decrease of the initiation of translation. However, this is negated by the fact that the cell cycle is also arrested by repression of translation elongation factors eEF3 (Figs 3 and 5). We also note that since cells are arrested in G1 (see below) and not in other cell cycle stages, the reduced translation capacity must have differential effects on the activity of proteins required for completing the different stages of the cell cycle.

Despite the similarity of the cell development responses to ribosomal and translational stress, the kinetics of the cell configuration changes is slower after cessation of eEF3 synthesis than after inhibition of r-protein synthesis (Fig 5). This could be because the translation capacity is not decreasing at equal rates in the two types of strains. However, another model is that the distortion of ribosomal assembly and the reduced translation capacity affect the cell cycle by independent mechanisms leading to a faster effect when both are at play while abrogating r-protein synthesis. Despite the clear effect of reducing eEF3, we can therefore not exclude that demolishing the ribosome assembly pathways also contributes after repressing r-protein gene expression as previously suggested [41, 43]. Thus ribosomal stress may affect the cell cycle by two different mechanisms.

### Cells are arrested in G1 between Ace2 migration and actin repolarization

Previous experiments pointed to arrest in both G1 and G2/M [39, 41]. Here we show that membrane ingression is complete (Figs 3 and 4) and transcription factor Ace2 continues to accumulate as the population of arrested cells builds up (Fig 6). Together these experiments clearly establish cells are arrested in G1 and proceeds at least beyond the localization of Ace2 to daughter nuclei. However, cell separation was delayed, as evidenced by accumulation of mother-daughter complexes (Figs 2–5). We note that ultimately 60–70% of the cells end up in mother-daughter complexes, while a much smaller fraction of the cells is in the flow cytometry 2N and 3N DNA peaks. One possible explanation for this difference is that the cell wall tethers, which hold the mother-daughter complexes together (Fig 6A(ii)), are broken by the forces of the laminar flow in the flow cytometer.

Our results also make it possible to narrow the stage in which cells are arrested within G1. The actin depolarization to budsites (Figs 7 and 8) suggests that cells are blocked before actin is repolarized, which takes place about 15 minutes prior to START [63, 64]. On the other hand, we observed that Ace2 is localized to daughter nuclei (Fig 6). We therefore conclude that the progress of cell cycle development is blocked between localization of Ace2 to daughter cell nuclei and the repolarization of actin patches, but not necessarily at actin repolarization, since actin depolarization appears to be delayed by one-two hours relative to the decline in the number of budded single cells. Actin polarization is believed to depend on activation of the Cdc42 GTPase by the complex of the Cdc28-Cln3 [17]. However, deletion of *CLN3* does not have any strong effect on the decreased budding during repression of the ribosome assembly factor Pwp2 [43] compatible with the notion that G1 progression is blocked even prior to Cdc42 activation.

Actin depolarization and ensuing cytoskeleton abnormality have also been observed during other kinds of stresses like oxidative, heat and cell wall stress [65, 66]. This commonality suggests that mechanisms activated during other forms of stress may also contribute to the ribosomal and translational stress response.

### No actin ring visible during mitosis or membrane ingression

The contraction of a ring of heavy myosin and actin is thought to be part of cell separation in organisms from yeast to humans [67, 68]. While we observed an actin ring in both mother and daughter cells before repression of r-protein genes, no ring was seen after about six hours of ribosomal stress (Figs 7 and 8). Cell division without an actin ring has also been observed in Δ*myo1* mutants. Deletion of *MYO1*, encoding heavy myosin, in *S*. *cerevisiae* is lethal, but mutations compromising function of the anaphase promoting complex suppress the lethality and enable cytokinesis without a visible actomyosin ring [68, 69]. Furthermore, cell separation is delayed and multiple septa and thick cell wall structures are formed between the mother and daughter cell in Δ*myo1* mutants [61, 69, 70]. The tethers binding mother and daughter cells together after mitosis during ribosomal stress (Fig 6) are potentially the result of such focused cell wall synthesis. Based on the similarity between cell division during ribosomal stress and in Δ*myo1* mutants, we propose that the cytokinesis and cell division may switch dynamically between actin ring-associated and actin ring-independent mode during ribosomal stress.

### Potential mechanisms connecting ribosome metabolism to cell cycle

The accumulation of mother-daughter complexes shows that cell separation after mitosis is delayed (Fig 5). Inactivation of the ribosome biogenesis factor SpNoc3 in *Schizosaccharomyces pombe* also inhibits normal cell separation, showing that the phenotype is not limited to *S*. *cerevisiae* [71]. The delayed cell separation suggests that expression of chitinases and gluconases, which promote cell separation, is reduced [56]. Indeed, measurements of mRNA abundance show significant reductions of mRNAs for *CTS1* (an endochitinase), *EGT2* (an endoglucanase), *DSE3* (a daughter cell-specific protein), and *DSE4* (a daughter protein with similarity to glucanases) (M. Shamsuzzaman, V. Bruno, and L. Lindahl, manuscript in preparation). Interestingly, transcription of CTS1 and EGT2 transcription is stimulated by both the Ace2 and Swi5 transcription factors [72, 73]. One or both of these transcription factors may thus contribute to the delayed cell separation.

Daughter cells in the mother-daughter complexes never (or rarely for eEF3 depletion) formed buds of their own, while mother cells often formed one more bud. Thus daughter cells are arrested before mother cells. A potential cause may be that daughter cells are insufficiently resourced to complete G1. Protein, mRNA, ribosomes and mitochondria are transported from mother to daughter cells along actin cables from mother cells to buds during unstressed growth. This includes 30 mRNAs [74–76], most of which encode proteins important for proper formation of membranes [77, 78]. This transport of resources to daughter cells may be reduced during ribosomal and translational stress, because of the hypodeveloped actin cytoskeleton.

Passage of cells from G1 to S phase is believed to require that cells have grown to a “critical size” [79]. However, small cell size does not appear to be the reason for G1 arrest during ribosomal arrest. Our measurement of forward light scattering shows that G1 cells are bigger during stress than the corresponding unstressed cells after the shift from galactose to glucose (S4 Fig and [41]). Using cell elutriation, Bernstein et al similarly found that stressed cells are bigger than control cells after depletion of Pwp2 [43]. The cause of G1 arrest can thus not be that the cells do not grow to the “critical size” required for passage of START. Something else is preventing budding, even though the cells have achieved “critical size” for budding.

The multifunctional phosphokinase TOR is part of two complexes, TORC1 and TORC2. Several properties of TORC2 are consistent with the notion that it has a role in the transition from uninhibited growth to cell cycle arrest. First, ribosomes associate with TORC2 and stimulate its activity [80]. Second, TORC2 controls actin polarity through a cascade involving the AGC kinases YPK1 and 2 [81, 82]. Third, TORC2 controls phosporylation of Crz1, a stress induced transcription factor, via a cascade involving Ypk1/2 and calcineurin [83, 84]. A decrease of TORC2 activity due to the decreased number of ribosomes would result decreased phosphorylation of Crz1 resulting in migration of Crz1 from the cytoplasm to the nucleus. This would stimulate transcription of several stress genes, including 1,3-beta-D-glucan synthase (GS) Fks2/Cgs2 [85], which could contribute to formation of the tether between mother and daughter cells (Fig 6).

## Conclusions and perspective

We have shown that decreased ribosomal translation rate by itself leads to G1 arrest. Reduced translation capacity is therefore a likely contributing factor to the cell cycle changes after reducing/abolishing ribosome formation. However, this does not exclude that dismantling of ribosomal assembly pathways contributes independently to the inhibition of cell cycle progression in G1. Importantly, changes of ribosome formation and function are sensed after a short time, raising the possibility that the mechanisms leading to cell cycle arrest also contribute to rectifying random fluctuations in ribosome synthesis or function in individual cells. Furthermore, the conservation of mechanisms for cell cycle, ribosome biogenesis, and ribosome function suggest that activation of stress-related genes in G1 phase, also could contribute to ribosomopathy diseases such as Blackfan Diamond Anemia. Interestingly, recent results suggest that ribosomopathy genes are also expressed during development [86], suggesting that mechanisms discovered during extreme ribosome related stress could also contribute to normal development.

## Supporting information

S1 Fig Growth curves. Strains were grown in galactose or shifted to glucose medium. (A) Pgal-eEF3: The TEF3 gene was placed on a plasmid and expressed from the gal promoter. The chromosomal TEF3 gene or both the TEF3 and HEF3 gene were deleted. (B) Pgal-eL43 and Pgal-uS4
(PDF)

S2 Fig Spindle dynamics after blocking synthesis of uL4. Pgal-uL4 was grown in galactose and shifted to glucose medium for 16 hours. The figure shows a merge of tub-GFP and brightfield images.
(PDF)

S3 Fig Mother cells with attached daughter cells after blocking the synthesis of uS4. Brightfield image of Pgal-uS4 16 hours after shift to glucose medium.
(PDF)

S4 Fig Cell size distribution after repression of r-protein genes. Flow cytometry (cell number vs. forward light scatter) of Pgal-uL4, -uL18, -eL43, and–uS4 growing in galactose or shifted to glucose for the indicated times.
(PDF)

S5 Fig Actin patch polarization after depletion of 40S ribosomal assembly factor Pwp2. Pgal-Pwp2 was grown in galactose medium and switched to glucose medium for 16 hours. Actin patches were stained with rhodamine-phalloidin. The figure shows merges of actin patches and brightfield images after growth in galactose (top) and glucose (bottom).
(PDF)

S6 Fig Actin patch polarization after depletion of 60S ribosomal assembly factor Nop7. Pgal-Nop7 was grown in galactose medium and switched to glucose medium for 16 hours. Actin patches were stained with rhodamine-phalloidin. The figure shows merges of actin patches and brightfield images after growth in galactose (left) and glucose (right).
(PDF)

S7 Fig Number of actin patches per cell. Pgal-eL43 and Pgal-eEF3 were grown in galactose and switched to glucose for 16 and 31 hours, respectively. Actin was stained with rhodamine-phalloidin, and finally the total number of actin patches was counted in different cells. Number of cells counted was 7 for Pgal-eL43 in galactose, 13 Pgal-eL43 in glucose, 3 for Pgal-eEF3 in galactose or glucose. The error bars indicate the standard error of the mean. Raw counts are available in S5 Table.
(PDF)

S1 Table Raw data for quantification of cell cycle developmental stages (graphed in Fig 5). Pgal-uS4, Pgal-eL43, and Pgal-eEF3 tagged with GFP-Ras2 and Spc42-RFP were grown in galactose medium and shifted to glucose medium for the indicated times. Cells were fixed and inspected by confocal microscopy. Cells were classified on field images according position of SPB and the completeness of the plasma membrane. Cells surrounded by a complete plasma membrane, indicating that cytokinesis was completed, were counted as individual cells, whether associated with other cells or not. The value for cell# indicates the number of cells in each type of mother-daughter complexes. “Total number of cells uncorrected” indicates the total number of raw counts. “Total number of cells corrected” is the sum of raw cell counts multiplies by the cell# value for each category.
(PDF)

S2 Table Raw data for zymolyase digestion of cell complexes graphed in Fig 6B. (PDF)

S3 Table Raw and derived numbers for quantification of actin images (graphed in Fig 8). Pgal-uS4, Pgal-eL43, and Pgal-eEF3 tagged with GFP-Ras2 were grown in galactose medium and shifted to glucose medium for the indicated times. Cells were stained with rhodamine-phalloidin and inspected by confocal microscopy. Cells were classified on field images and quantified. We counted each cells with a complete plasma membrane as an individual cell. Furthermore, cells were classified depending on the distribution of actin patches. Classified raw counts of cells or mother-daughter complexes are written in black. Each category of mothers with buds and mother-daughter complexes was then parsed according to the actin distribution in each cell within free cells and complexes. Note that categories 13–15 were not found after repression of the uS4 or eL43 genes. The right side of S3 Table shows calculations of the aggregate number of cells in which actin patches were polarized to buds/budsites or budnecks, or in which actin patches were dispersed to the cell cortex. The blue-shaded columns show the data plotted in Fig 8.
(PDF)

S4 Table Structure of categories used in S3 Table. Bin numbers and hand-drawn sketches of typical cell configurations in each category. Also shown are the weights for distribution of cells into aggregate categories.
(PDF)

S5 Table Counts of actin patches per cell. Details for strains and growth are in the legend to S7 Fig. Data from this table are graphed in S7 Fig.
(PDF)
