# Supplementary figures and images for: Analysis of cell cycle parameters during the transition from unhindered growth to ribosomal and translational stress conditions
Source: PLoS One. 2017 Oct 13;12(10):e0186494. doi: 10.1371/journal.pone.0186494 (PMC5640253; doi:10.1371/journal.pone.0186494)

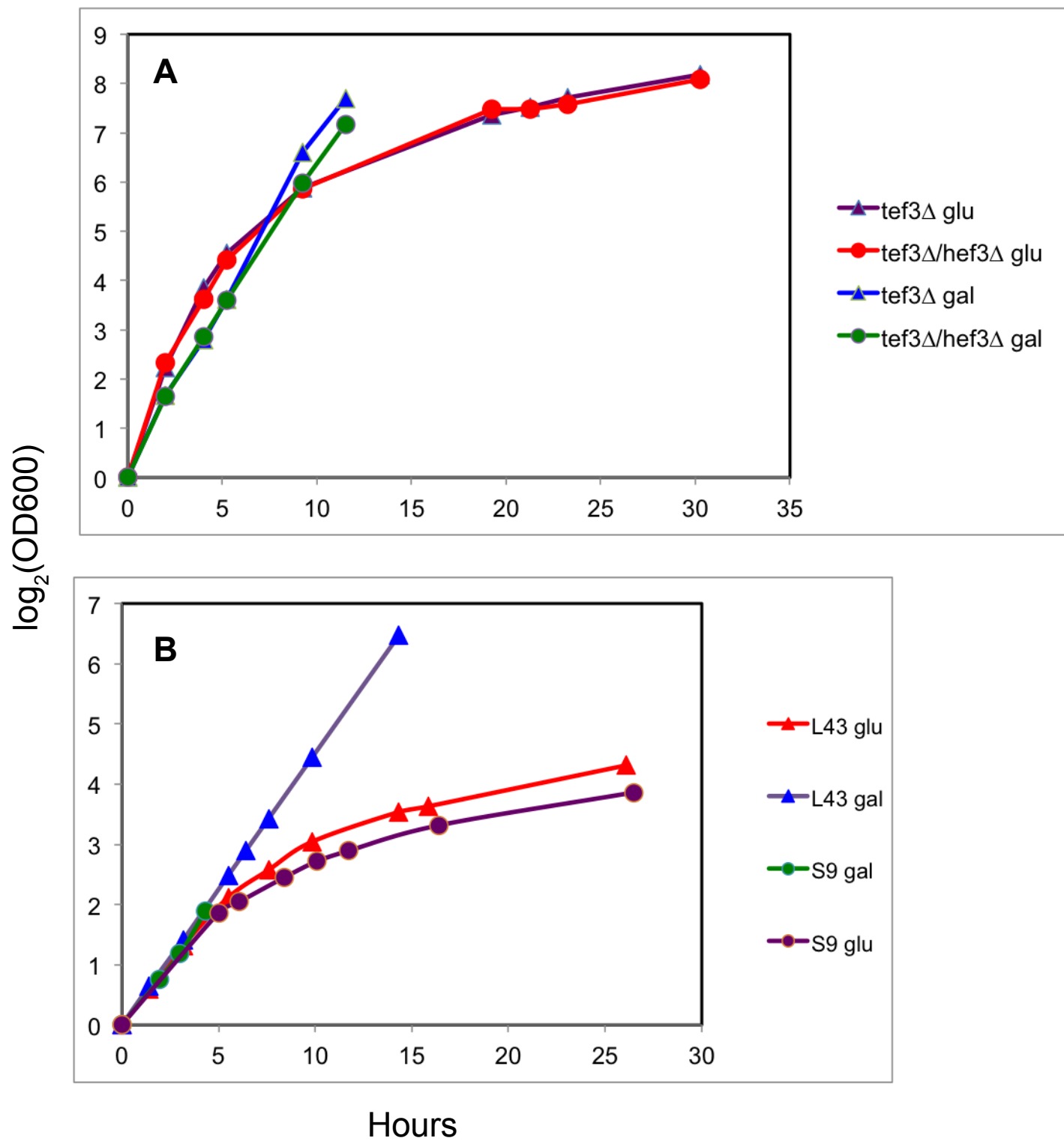

Fig S1

Supplement: S1 Fig — Strains were grown in galactose or shifted to glucose medium. (A) Pgal-eEF3: The TEF3 gene was placed on a plasmid and expressed from the gal promoter. The chromosomal TEF3 gene or both the TEF3 and HEF3 gene were deleted. (B) Pgal-eL43 and Pgal-uS4 (PDF) [file pone.0186494.s001.pdf]

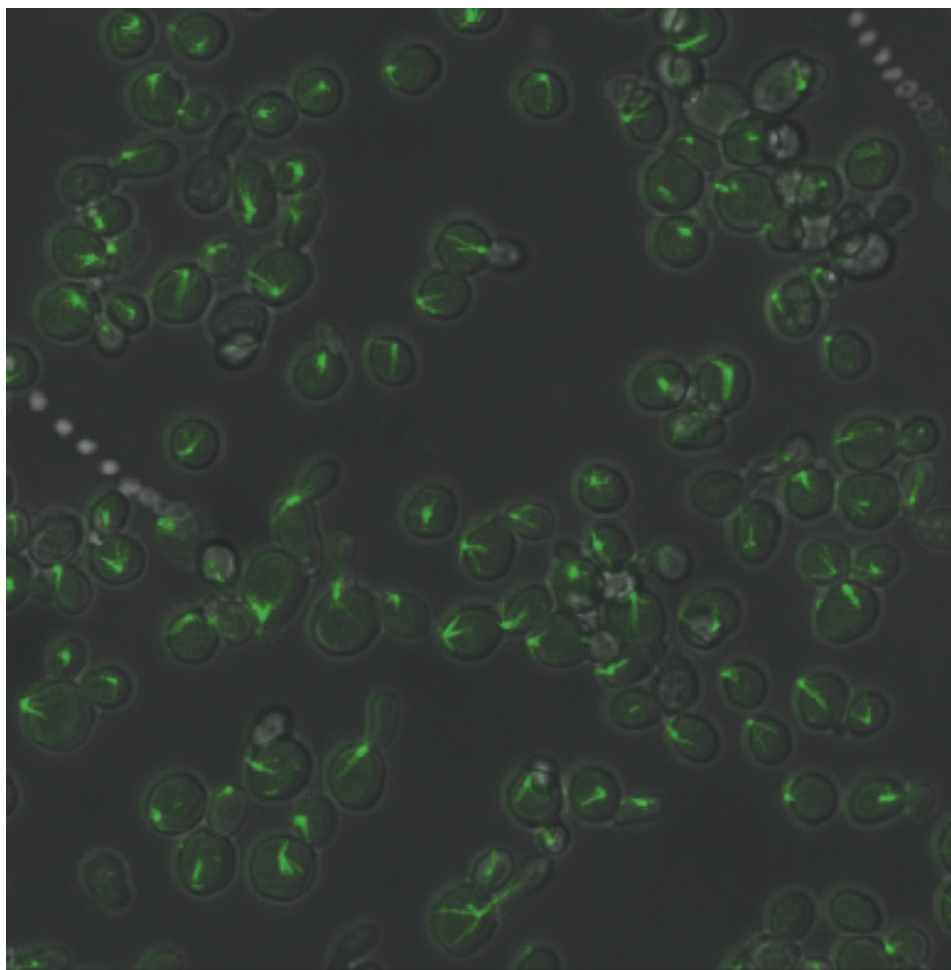

Tub-GFP after repressing uL4 synthesis for 16 hours

Fig S2

Supplement: S2 Fig — Pgal-uL4 was grown in galactose and shifted to glucose medium for 16 hours. The figure shows a merge of tub-GFP and brightfield images. (PDF) [file pone.0186494.s002.pdf]

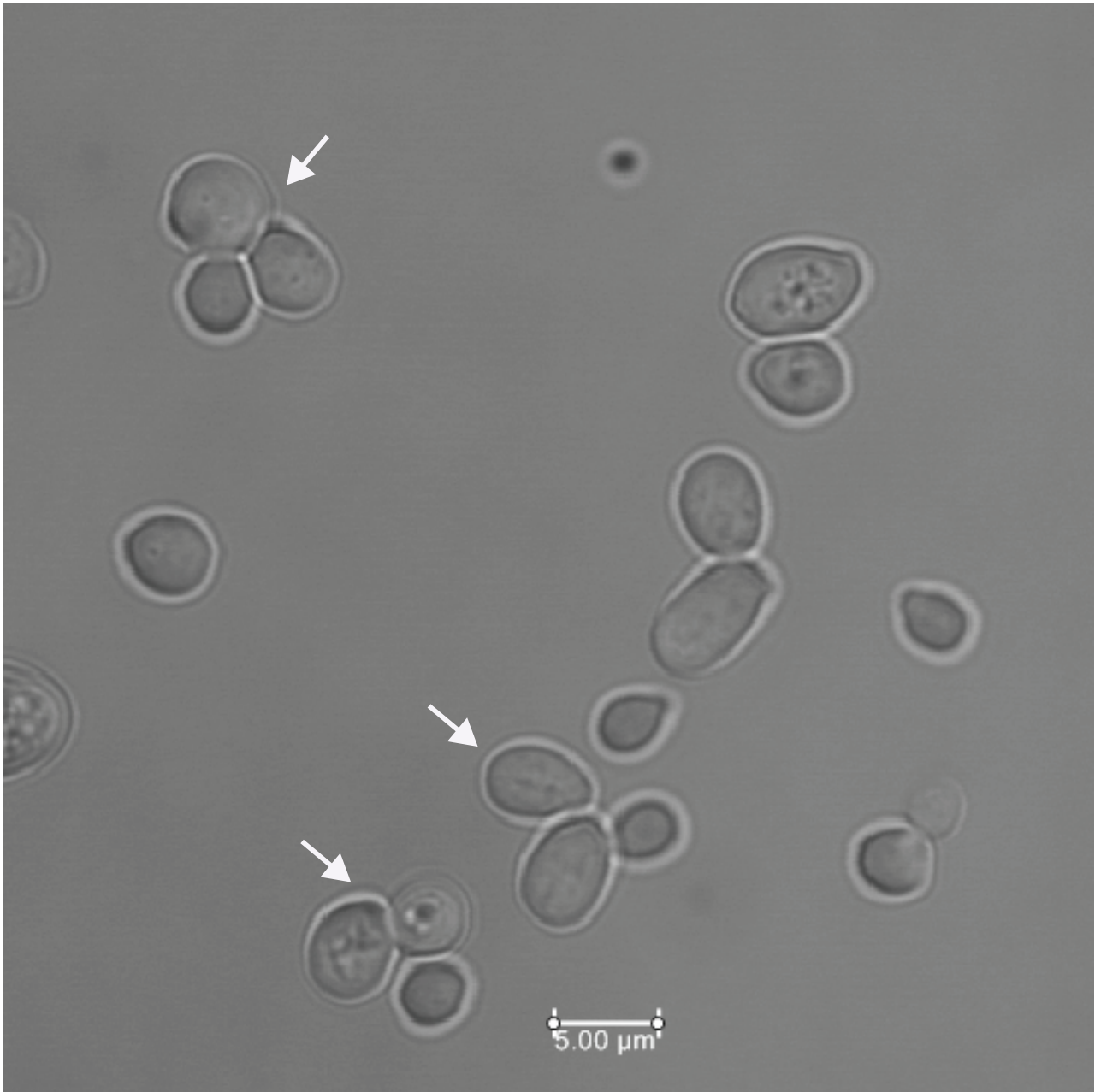

Pgal-uS4 after 16 hours in glucose medium

Supplement: S3 Fig — Brightfield image of Pgal-uS4 16 hours after shift to glucose medium. (PDF) [file pone.0186494.s003.pdf]

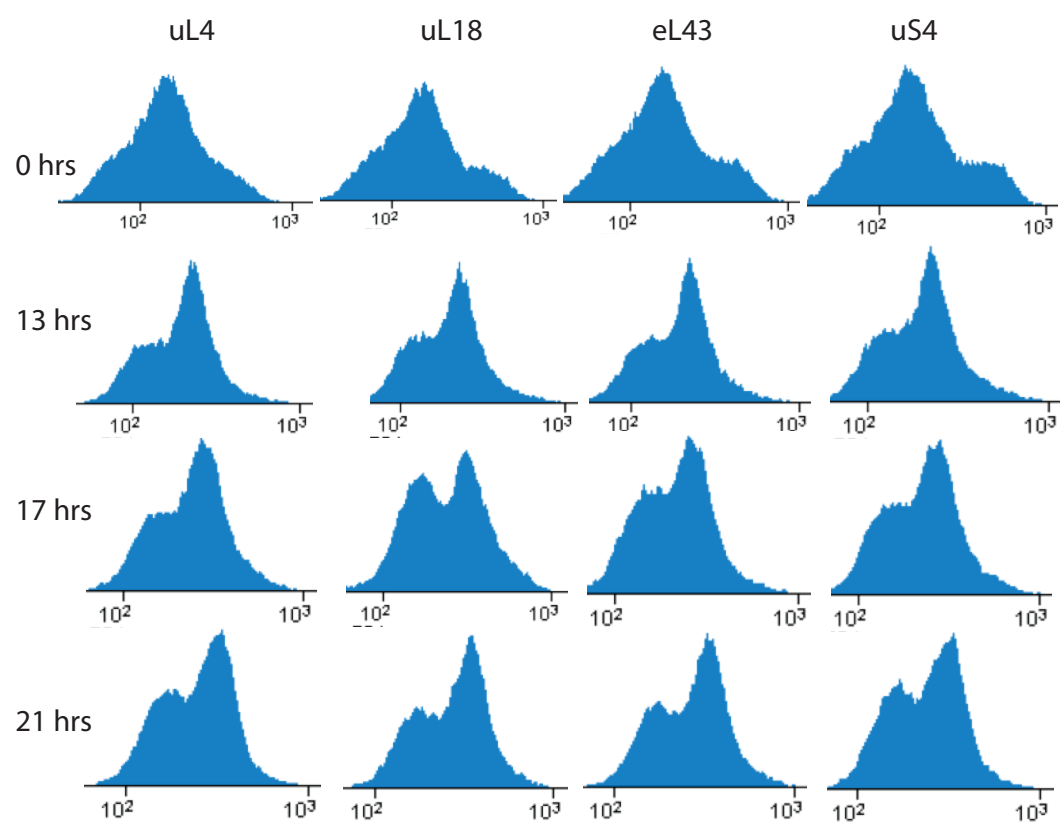

Fig S4

Supplement: S4 Fig — Flow cytometry (cell number vs. forward light scatter) of Pgal-uL4, -uL18, -eL43, and–uS4 growing in galactose or shifted to glucose for the indicated times. (PDF) [file pone.0186494.s004.pdf]

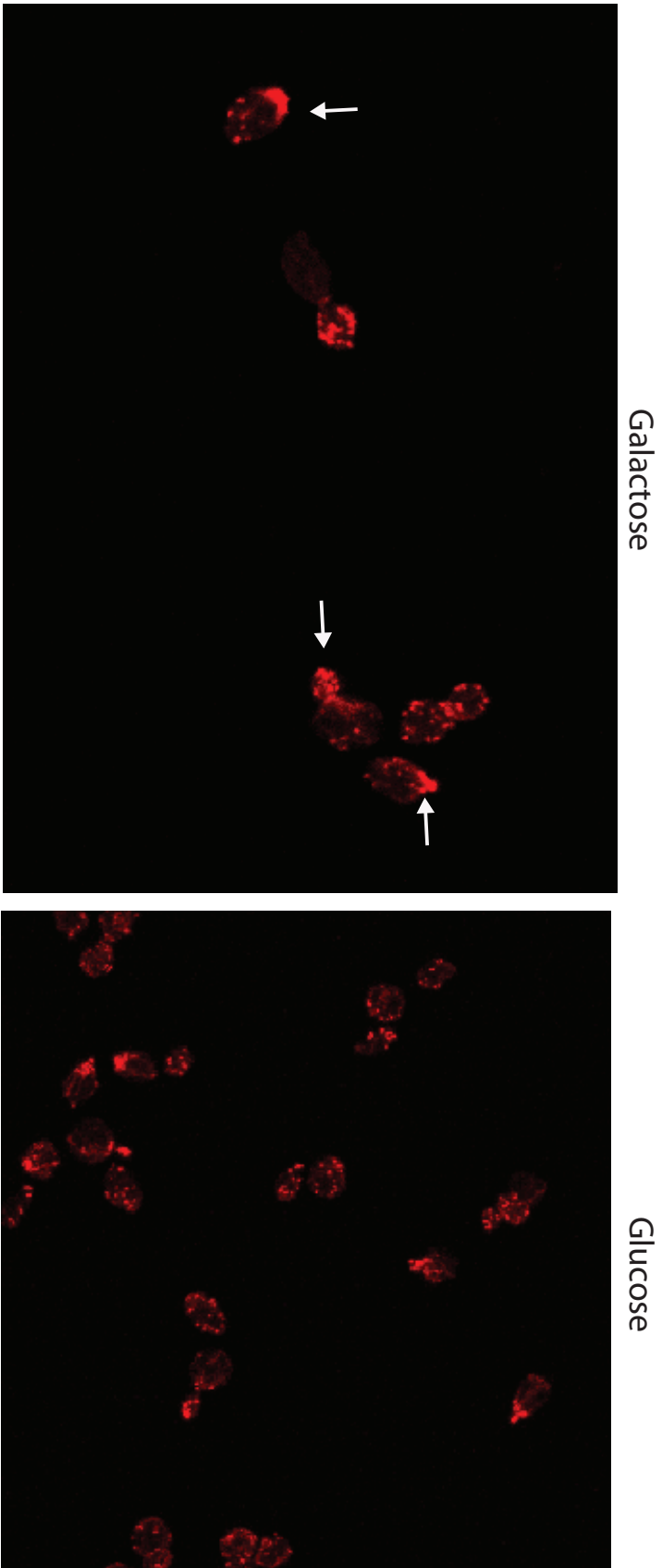

Pwp2  
Fig S5

Supplement: S5 Fig — Pgal-Pwp2 was grown in galactose medium and switched to glucose medium for 16 hours. Actin patches were stained with rhodamine-phalloidin. The figure shows merges of actin patches and brightfield images after growth in galactose (top) and glucose (bottom). (PDF) [file pone.0186494.s005.pdf]

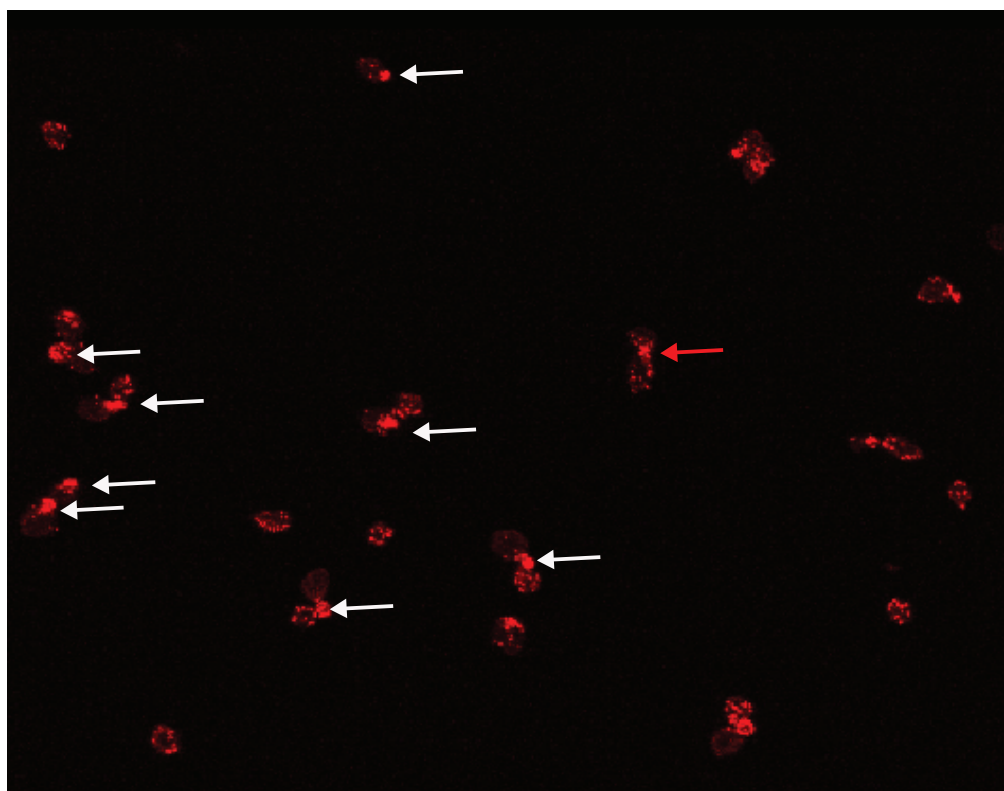

Galactose

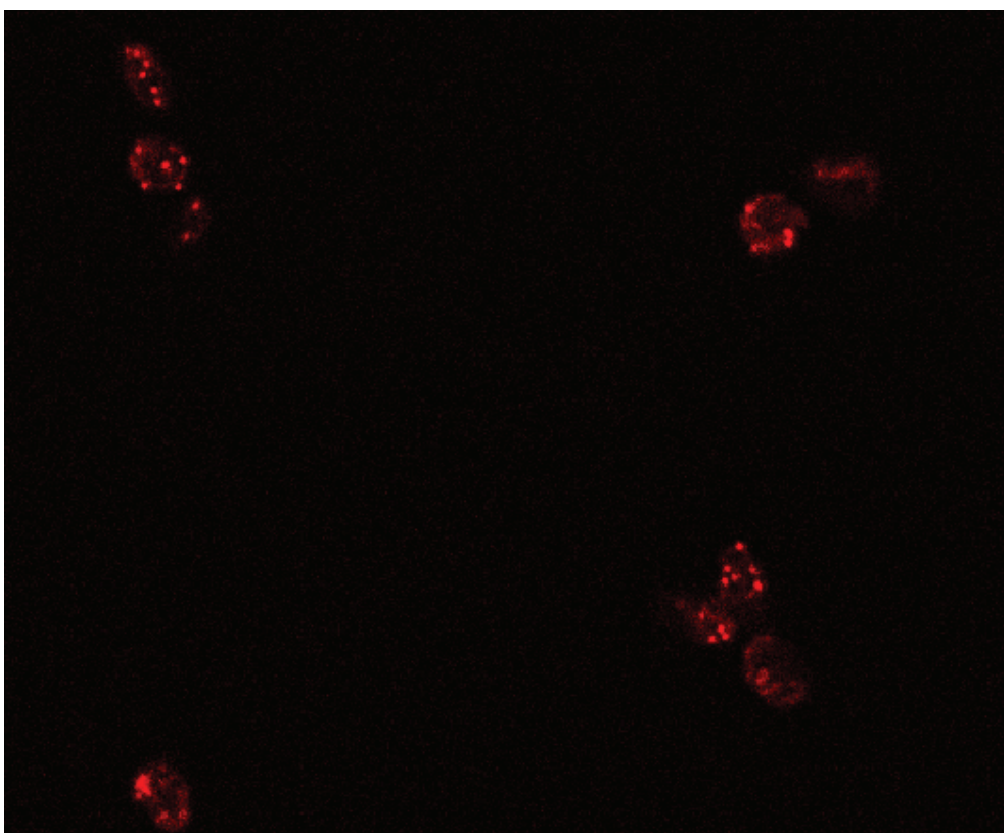

Glucose

Nop7

Fig S6

Supplement: S6 Fig — Pgal-Nop7 was grown in galactose medium and switched to glucose medium for 16 hours. Actin patches were stained with rhodamine-phalloidin. The figure shows merges of actin patches and brightfield images after growth in galactose (left) and glucose (right). (PDF) [file pone.0186494.s006.pdf]

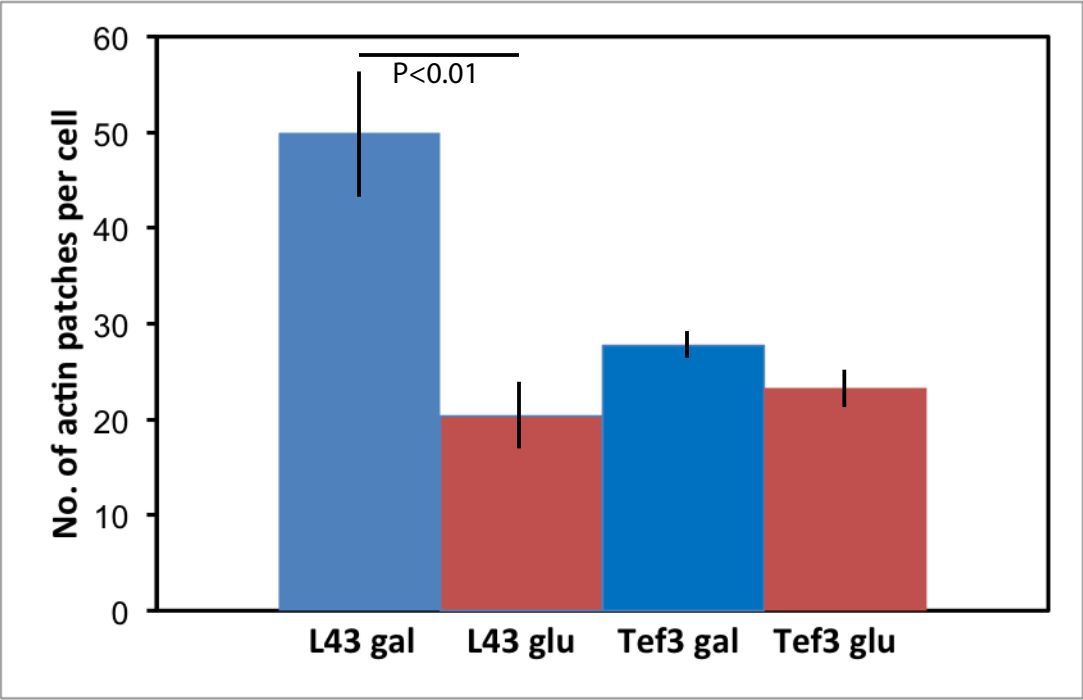

Fig S7

Supplement: S7 Fig — Pgal-eL43 and Pgal-eEF3 were grown in galactose and switched to glucose for 16 and 31 hours, respectively. Actin was stained with rhodamine-phalloidin, and finally the total number of actin patches was counted in different cells. Number of cells counted was 7 for Pgal-eL43 in galactose, 13 Pgal-eL43 in glucose, 3 for Pgal-eEF3 in galactose or glucose. The error bars indicate the standard error of the mean. Raw counts are available in S5 Table. (PDF) [file pone.0186494.s007.pdf]
