# Supplementary material for: Analysis of cell cycle parameters during the transition from unhindered growth to ribosomal and translational stress conditions
Source: PLoS One. 2017 Oct 13;12(10):e0186494. doi: 10.1371/journal.pone.0186494 (PMC5640253; doi:10.1371/journal.pone.0186494)
Supplement: S1 Table — Pgal-uS4, Pgal-eL43, and Pgal-eEF3 tagged with GFP-Ras2 and Spc42-RFP were grown in galactose medium and shifted to glucose medium for the indicated times. Cells were fixed and inspected by confocal microscopy. Cells were classified on field images according position of SPB and the completeness of the plasma membrane. Cells surrounded by a complete plasma membrane, indicating that cytokinesis was completed, were counted as individual cells, whether associated with other cells or not. The value for cell# indicates the number of cells in each type of mother-daughter complexes. “Total number of cells uncorrected” indicates the total number of raw counts. “Total number of cells corrected” is the sum of raw cell counts multiplies by the cell# value for each category. (PDF) [file pone.0186494.s008.pdf]

Strain	Time	Single cell - 1 SPB	Single cell - 1 SPB - small bud	Single cell - 1 SPB - large bud	Single cell with bud - 2 SPBs - not near bud neck	Single cell with bud - 2 SPBs - 1 in mother cell and 1 in budneck	Single cell with bud - 2 SPBs - shorter spindle than anaphase	Single cell with bud - 2 SPBs- medium size spindle	Single cell with bud - 2 SPBs at oppsite ends of mother and daughter cells - like anaphase	2 attached cells enclosed by membrane - 2 SPBs	2 attached cells enclosed by membrane - smother has a small bud - 2 SPBs	2 attached cells - mother has bud - mother, daughter and bud each have 1 SPB	2 attached cells- mother has bud - mother, daughter and bud each have 1 SPB - SPBs in mother and bud make a spindle	3 attached cells separated by membrane - 1 SPB in each cell	3 attached cells separated by membrane - 1 SPB in each cell - one of the 3 cells has a small bud	4 SPB	Total number of cells uncorrected	Total number corrected
		1	2	3	4	5	6	6.5	7	8	9	10	10a	11	12	13		
	Cell #	1	1	1	1	1	1	1	1	2	2	2	2	3	3	4		
Pgal-uS4																		
	0	36	6	2	15	14	9	5	7	7	0	0	0	0	0	0	101	108
	1	107	9	6	26	48	37	14	20	8	0	6	0	0	0	0	281	295
	2	68	15	6	18	48	31	9	12	12	1	5	0	0	0	0	225	243
	4	40	1	3	8	22	10	2	5	4	0	1	0	0	0	0	96	101
	8	93	0	0	10	20	3	6	2	13	0	6	0	0	0	0	153	172
	16	60	0	0	1	7	3	3	0	22	1	5	0	2	0	0	104	136
Pgal-eL43																		
experiment 1	0	101	26	15	18	30	20		16	15	0	0	0	0	0	0	241	256
	3	78	19	2	34	45	29		10	14	3	4	0	0	0	0	238	259
	6	113	11	1	16	37	14		3	24	3	4	0	2	0	0	228	263
	9	37	3	0	2	6	12		2	26	3	3	0	2	0	0	96	132
	12	86	9	0	6	25	22		3	48	8	15	0	3	0	0	225	302
	16	64	0	0	3	16	7		0	54	6	18	0	16	0	0	184	294
Pgal-eL43																		
Experiment 2	0	75	11	2	25	25	17		20	4	0	0	0	0	0	0	179	183
	1	89	19	8	20	29	38		12	10	0	0	0	0	0	0	225	235
	2	98	10	7	53	83	56		22	13	0	3	0	0	0	0	345	361
	4	83	5	4	12	36	26		2	7	3	3	0	0	0	0	181	194
	8	138	4	1	22	42	19		10	32	4	8	0	2	0	0	282	330
	16	107	2	0	2	11	11		3	38	2	10	0	5	0	0	191	251
Pgal-eEF1																		
Experiment 1	0	30	20	8	15	16	16		19	2	0	3	0	0	0	0	129	134
	4	33	8	2	12	28	14		12	12	8	0	0	0	0	0	129	149
	9	35	10	1	21	23	9		5	11	2	1	0	0	0	0	118	132
	13	66	5	3	31	14	9		4	19	3	3	0	0	0	0	157	182
	20	15	2	0	3	0	3		3	17	2	2	6	7	1	0	61	104
	31	35	0	1	5	2	0		0	25	0	3	7	14	4	1	97	171
Pgal-eEF1																		
Experiment 2	0	46	15	7	11	13	7		5	2	0	0	0	0	0	0	106	108
	9	28	7	0	7	11	7		3	6	2	0	1	0	0	0	72	81
	20	25	1	0	2	2	5		3	20	0	2	1	7	0	1	69	109
	31	61	0	0	6	3	4		4	29	2	2	5	9	1	2	128	192
