# Supplementary material for: Analysis of cell cycle parameters during the transition from unhindered growth to ribosomal and translational stress conditions
Source: PLoS One. 2017 Oct 13;12(10):e0186494. doi: 10.1371/journal.pone.0186494 (PMC5640253; doi:10.1371/journal.pone.0186494)
Supplement: S2 Table — (PDF) [file pone.0186494.s009.pdf]

Table S2. Count of budded cells after zymolyase digestion

	Pgal eL43		Cell count	Distribution (%)	Pgal-uL30	Distributio n (%)
Glucose	digested	Unbudded	245	87.5	711	81.6
		Single bud	32	11.4	144	16.5
		Dibuds	3	1.1	16	1.8
		Total	280		871	
	Undigested	Unbudded	82	43.2	165	40.9
		Single bud	74	38.9	149	37.0
		Dibuds	34	17.9	89	22.1
		Total	190		403	
Galactose	Digested	Unbudded	96	59.6		
		Single bud	65	40.4		
		Dibuds	0	0.0		
		Total	161			
	Undigested	Unbudded	73	42.0		
		Single bud	99	56.9		
		Dibuds	2	1.1		
		Total	174			
