# Supplementary material for: Analysis of cell cycle parameters during the transition from unhindered growth to ribosomal and translational stress conditions
Source: PLoS One. 2017 Oct 13;12(10):e0186494. doi: 10.1371/journal.pone.0186494 (PMC5640253; doi:10.1371/journal.pone.0186494)
Supplement: S3 Table — Pgal-uS4, Pgal-eL43, and Pgal-eEF3 tagged with GFP-Ras2 were grown in galactose medium and shifted to glucose medium for the indicated times. Cells were stained with rhodamine-phalloidin and inspected by confocal microscopy. Cells were classified on field images and quantified. We counted each cells with a complete plasma membrane as an individual cell. Furthermore, cells were classified depending on the distribution of actin patches. Classified raw counts of cells or mother-daughter complexes are written in black. Each category of mothers with buds and mother-daughter complexes was then parsed according to the actin distribution in each cell within free cells and complexes. Note that categories 13–15 were not found after repression of the uS4 or eL43 genes. The right side of S3 Table shows calculations of the aggregate number of cells in which actin patches were polarized to buds/budsites or budnecks, or in which actin patches were dispersed to the cell cortex. The blue-shaded columns show the data plotted in Fig 8. (PDF) [file pone.0186494.s010.pdf]

Table S3. Raw and processed data for actin polarity

Descriptive category	No bud - Not Polarized				No bud - Polarized				Small bud - polarized to the tip				Medium bud - polarized to the tip				Large bud - mostly dispersed in the bud cortex or polarized to the bud tip				Before cytokinesis - Dispersed in entire cell cortex				Polarized to bud neck				Incomplete or faulty polarization to bud neck					After cytokinesis - Dispersed in entire cell cortex			
Category #	1				2				3				4				5				6				7				7.5					8			
Pgal-el43																																					
Time (hrs)	Bud site	Budneck	Dispersed	Total	Bud site	Budneck	Dispersed	Total	Bud site	Budneck	Dispersed	Total	Bud site	Budneck	Dispersed	Total	Bud site	Budneck	Dispersed	Total	Bud site	Budneck	Dispersed	Total	Bud site	Budneck	Dispersed	Total	Bud site	Budneck	Dispersed	Total		Bud site	Budneck	Dispersed	Total
0	27				32				37				41				12				25				12				0					3			
	0	0	27	27	32	0	0	32	37	0	0	37	41	0	0	41	12	0	0	12	0	0	25	25	0	24	0	24	0	0	0	0		0	0	6	6
1	16				47				30				13				22				29				12				0					4			
	0	0	16	16	47	0	0	47	30	0	0	30	13	0	0	13	22	0	0	22	0	0	29	29	0	24	0	24	0	0	0	0		0	0	8	8
2	5				15				37				50				45				47				7				7					5			
	0	0	5	5	15	0	0	15	37	0	0	37	50	0	0	50	45	0	0	45	0	0	47	47	0	14	0	14	0	14	0	14		0	0	10	10
4	49				44				35				35				30				39				9				9					5			
	0	0	49	49	44	0	0	44	35	0	0	35	35	0	0	35	30	0	0	30	0	0	39	39	0	18	0	18	0	18	0	18		0	0	10	10
8	13				5				3				8				8				6				2				4					8			
	0	0	13	13	5	0	0	5	3	0	0	3	8	0	0	8	8	0	0	8	0	0	6	6	0	4	0	4	0	8	0	8		0	0	16	16
16	105				2				4				5				13				3				0				3					54			
	0	0	105	105	2	0	0	2	4	0	0	4	5	0	0	5	13	0	0	13	0	0	3	3	0	0	0	0	0	6	0	6		0	0	108	108
Pgal-uS4																																					
0	15				18				10				7				10				13				0				5					1			
	0	0	15	15	18	0	0	18	10	0	0	10	7	0	0	7	10	0	0	10	0	0	13	13	0	0	0	0	0	10	0	10		0	0	2	2
4	61				16				13				6				28				34				7				8					11			
	0	0	61	61	16	0	0	16	13	0	0	13	6	0	0	6	28	0	0	28	0	0	34	34	0	14	0	14	0	16	0	16		0	0	22	22
8	57				7				1				4				11				18				3				1					18			
	0	0	57	57	7	0	0	7	1	0	0	1	4	0	0	4	11	0	0	11	0	0	18	18	0	6	0	6	0	2	0	2		0	0	36	36
16	71				1				2				0				7				10				0				0					25			
	0	0	71	71	1	0	0	1	2	0	0	2	0	0	0	0	7	0	0	7	0	0	10	10	0	0	0	0	0	0	0	0		0	0	50	50
Pgal-eEF3																																					
Exp1	Bud site	Budneck	Dispersed	Total	Bud site	Budneck	Dispersed	Total	Bud site	Budneck	Dispersed	Total	Bud site	Budneck	Dispersed	Total	Bud site	Budneck	Dispersed	Total	Bud site	Budneck	Dispersed	Total	Bud site	Budneck	Dispersed	Total	Bud site	Budneck	Dispersed	Total		Bud site	Budneck	Dispersed	Total
0	45				92				59				80				58				33				36				0					0			
	0	0	45	45	92	0	0	92	59	0	0	59	80	0	0	80	58	0	0	58	0	0	33	33	0	72	0	72	0	0	0	0		0	0	0	0
5	42				90				46				55				59				9				33				0					0			
	0	0	42	42	90	0	0	90	46	0	0	46	55	0	0	55	59	0	0	59	0	0	9	9	0	66	0	66	0	0	0	0		0	0	0	0
10	70				72				36				36				39				8				36				0					8			
	0	0	70	70	72	0	0	72	36	0	0	36	36	0	0	36	39	0	0	39	0	0	8	8	0	72	0	72	0	0	0	0		0	0	16	16
28	29				14				2				10				2				2				4				0					10			
	0	0	29	29	14	0	0	14	2	0	0	2	10	0	0	10	2	0	0	2	0	0	2	2	0	8	0	8	0	0	0	0		0	0	20	20
Pgal-eEF3																																					
Exp2																																					
0	14				9				0				7				11				7				4				0					1			
	0	0	14	14	9	0	0	9	0	0	0	0	7	0	0	7	11	0	0	11	0	0	7	7	0	8	0	8		0	0	0		0	0	2	2
31	22				5				1				4				5				3				1				0					11			
	0	0	22	22	5	0	0	5	1	0	0	1	4	0	0	4	5	0	0	5	0	0	3	3	0	2	0	2		0	0	0		0	0	22	22

1 daughter & 1 small bud - polarization to bud tip - dispersed in daughter				1 daughter & 1 small bud - polarization to bud tip - dispersed in daughter				2 daughters - Dispersed in mother & in both daughters				Three connected cells. There is actin ring between two of these cells				3 connectes, but seoparated cells, and one of these cells is polarized to budsite				Four connectes, but separated cells. In some cases one of these cells may have polarization				Time		Total cells	All cells per category				All cells per category normalized to total cell number				Descriptive category
9				10				11				13				14				15															Category #
																																			Pgal-el43
Bud site	Budneck	Dispersed	Total	Bud site	Budneck	Dispersed	Total	Bud site	Budneck	Dispersed	Total	Bud site	Budneck	Dispersed	Total	Bud site	Budneck	Dispersed	Total	Bud site	Budneck	Dispersed	Total		Total corrected		Bud site	Budneck	Dispersed	Total	Bud site	Budneck	Dispersed	Total	Time (hrs)
0				0				0																0		189									0
0	0	0	0	0	0	0	0	0	0	0	0														204		122	24	58	204	0.60	0.12	0.28	1.00	
5				2				2																1		182									1
5	0	5	10	1	0	3	4	0	0	6	6														209		118	24	67	209	0.56	0.11	0.32	1.00	
2				1				0																2		221									2
2	0	2	4	0.5	0	1.5	2	0	0	0	0														243		149.5	28	65.5	243	0.62	0.12	0.27	1.00	
6				2				1																4		264									4
6	0	6	12	1	0	3	4	0	0	3	3														297		151	36	110	297	0.51	0.12	0.37	1.00	
1				1				1																8		60									8
1	0	1	2	0.5	0	1.5	2	0	0	3	3														78		25.5	12	40.5	78	0.33	0.15	0.52	1.00	
1				18				14																16		222									16
1	0	1	2	9	0	27	36	0	0	42	42														326		34	6	286	326	0.10	0.02	0.88	1.00	
																																			Pgal-uS4
0				0				0																0		79									0
0	0	0	0	0	0	0	0	0	0	0	0														85		45	10	30	85	0.53	0.12	0.35	1.00	
3				2				0																4		189									4
3	0	3	6	1	0	3	4	0	0	0	0														220		67	30	123	220	0.30	0.14	0.56	1.00	
1				3				3																8		127									8
1	0	1	2	1.5	0	4.5	6	0	0	9	9														159		25.5	8	125.5	159	0.16	0.05	0.79	1.00	
2				3				2																16		123									16
2	0	2	4	1.5	0	4.5	6	0	0	6	6														157		13.5	0	143.5	157	0.09	0.00	0.91	1.00	
																								Tef3											Pgal-eEF3
Bud site	Budneck	Dispersed	Total	Bud site	Budneck	Dispersed	Total	Bud site	Budneck	Dispersed	Total	Bud site	Budneck	Dispersed	Total	Bud site	Budneck	Dispersed	Total	Bud site	Budneck	Dispersed	Total		Total correct ed		Bud site	Budneck	Dispersed	Total	Bud site	Budneck	Dispersed	Total	Exp1
3				0				0				0				0				0				0		406									0
3	0	3	6	0	0	0	0	0	0	0	0	0	0	0	0	0	0	0	0	0	0	0	0		445		292	72	81	445	0.66	0.16	0.18	1.00	
2				0				0				0				0				0				5		336									5
2	0	2	4	0	0	0	0	0	0	0	0	0	0	0	0	0	0	0	0	0	0	0	0		371		252	66	53	371	0.68	0.18	0.14	1.00	
13				9				0				0				0				0				10		327									10
13	0	13	26	9	0	9	18	0	0	0	0	0	0	0	0	0	0	0	0	0	0	0	0		393		205	72	116	393	0.52	0.18	0.30	1.00	
6				9				8				5				23				9				28		133									28
6	0	6	12	9	0	9	18	0	0	24	24	0	10	5	15	23	0	46	69	4.5	0	31.5	36		261		70.5	18	172.5	261	0.27	0.07	0.66	1.00	
																																			Pgal-eEF3
																																			Exp2
0				0				0																0											0
0	0	0	0	0	0	0	0	0	0	0	0	0	0	0	0	0	0	0	0	0	0	0	0		58		27	8	23	58	0.47	0.14	0.40	1.00	
8				7				8				2				2				4				31											31
8	0	8	16	7	0	7	14	0	0	24	24	0	4	2	6	2	0	4	6	2	0	14	16		146		34	6	106	146	0.23	0.04	0.73	1.00	
