# Supplementary material for: Analysis of cell cycle parameters during the transition from unhindered growth to ribosomal and translational stress conditions
Source: PLoS One. 2017 Oct 13;12(10):e0186494. doi: 10.1371/journal.pone.0186494 (PMC5640253; doi:10.1371/journal.pone.0186494)
Supplement: S4 Table — Bin numbers and hand-drawn sketches of typical cell configurations in each category. Also shown are the weights for distribution of cells into aggregate categories. (PDF) [file pone.0186494.s011.pdf]

Table S4. Categories actin images and their caracterization

Category	Sketch	Distribution				
		Polarized to budsite	Polarized to budneck	Disperse	Number of cells	Description
1	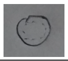	0	0	1	1	No bud - Not Polarized
2	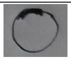	1	0	0	1	No bud - Polarized
3	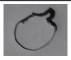	1	0	0	1	Small bud - polarized to the tip
4	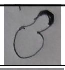	1	0	0	1	Medium bud - polarized to the tip
5	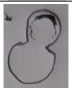	1	0	0	1	Large bud - mostly dispersed in the bud cortex or polarized to the bud tip
6	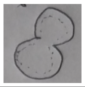	0	0	1	1	Before cytokinesis - Dispersed in entire cell cortex
7	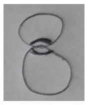	0	2	0	2	Polarized to bud neck
7.5	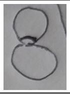	0	1	0	1	Incomplete or faulty polarization to bud neck
8	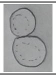	0	0	2	2	After cytokinesis - Dispersed in entire cell cortex
9	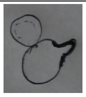	1	0	1	2	1 daughter & 1 small bud - polarization to bud tip - dispersed in daughter
10 (two types difficult to distinguish)	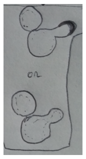	0.5	0	1.5	2	1 daughter & 1 small bud - polarization to bud tip - dispersed in
11	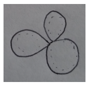	0	0	3	3	2 daughters - Dispersed in mother & in both daughters
13	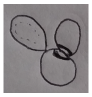	0	2	1	3	Three connected cells. There is actin ring between two of these cells
14 (three types difficult to distinguish)	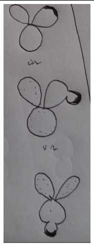	1	0	2	3	Three connected but separated cells, and one of these cells is polarized or formed bud
15 (two types difficult to distinguish)	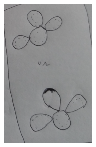	0.5	0	3.5	4	Four connected but separated cells. In some cases any of these cells may have polarization
