# Supplementary material for: Analysis of cell cycle parameters during the transition from unhindered growth to ribosomal and translational stress conditions
Source: PLoS One. 2017 Oct 13;12(10):e0186494. doi: 10.1371/journal.pone.0186494 (PMC5640253; doi:10.1371/journal.pone.0186494)
Supplement: S5 Table — Details for strains and growth are in the legend to S7 Fig. Data from this table are graphed in S7 Fig. (PDF) [file pone.0186494.s012.pdf]

Table S6. Actin patches per cell

Strain	Medium	Cell	Actin patches Mother	Daughter	Recount	Average
Pgal-eL43	Galactose	1				
		2		27	24	25.5
		3	58		50	54.0
		4	49		45	47.0
		5	35		30	32.5
		6	84		72	78.0
		7		64	61	62.5
		8	55		46	50.5
	Mean					50.0
	Standard deviation					17.7
	Standard error					6.3
Pgal-eL43	Glucose (16 hrs)	1	14			14.0
		2		9		9.0
		3	31		28	29.5
		4	28		25	26.5
		5	13		13	13.0
		6		17	28	22.5
		7	22		23	22.5
		8	20		15	17.5
		9	40		34	37.0
		10	39		35	37.0
		11	14		19	16.5
		12		3	3	3.0
		13	13		20	16.5
	Mean					20.3
	Standard deviation					10.2
	Standard error					2.8
Pgal-eEF3	Galactose	1	15	12		13.5
		2	16	14		15.0
		3	16	13		14.5
	Mean					14.3
	Standard deviation					0.8
	Standard error					0.4
Pgal-eEF3	Glucose (31 hrs)	1	17	8		12.5

	2	13	10	11.5
	3	14	8	11.0
Mean				11.7
Standard deviation				0.8
Standard error				0.4
